# Highly neurogenic glia from human and mouse myenteric ganglia generate functional neurons following culture and transplantation into the gut

**DOI:** 10.1016/j.celrep.2024.114919

**Published:** 2024-10-30

**Authors:** Jessica L. Mueller, Abigail R. Leavitt, Ahmed A. Rahman, Christopher Y. Han, Leah C. Ott, Narges S. Mahdavian, Simona E. Carbone, Sebastian K. King, Alan J. Burns, Daniel P. Poole, Ryo Hotta, Allan M. Goldstein, Rhian Stavely

**Affiliations:** 1 Department of Pediatric Surgery, Massachusetts General Hospital, Harvard Medical School, Boston, MA, USA; 2 Drug Discovery Biology, Monash Institute of Pharmaceutical Sciences, Monash University, Parkville, VIC, Australia; 3 Department of Paediatric Surgery, The Royal Children’s Hospital, Parkville, VIC, Australia; 4 Lead contact

## Abstract

Enteric neural stem cell (ENSC) therapy offers great promise for neurointestinal diseases; however, current isolation methods yield insufficient neurons for regenerative applications. Multiomic profiling of enteric glial cells (EGCs) suggests that subpopulations within myenteric ganglia (MyGa) are a reservoir of highly neurogenic ENSCs. Here, we describe protocols to enrich for intraganglionic EGCs by isolating intact fragments of MyGa, generating cultures with higher neuronal purity than traditional methodologies isolating intramuscular single cells (IM-SCs). MyGa-derived EGCs transdifferentiate into more neurons than IM-SC-derived EGCs do, confirming their neurogenic predisposition. Following transplantation to the mouse intestine, MyGa-derived neurons generate calcium transients and activate smooth muscle in response to optogenetic stimulation. In the human intestine, MyGa-derived cells are similarly highly neurogenic, are enriched for a distinct progenitor population identified by single-cell RNA sequencing (scRNA-seq), and exhibit neuromuscular connectivity following xenogeneic transplantation into mice. Highly neurogenic ENSCs are preferentially located within the MyGa, and their selective isolation offers considerable potential for therapy.

## In brief

Mueller et al. show that selectively isolated ENSCs from MyGa have a high neuronal differentiation potential. These findings are leveraged to develop protocols to generate ENSC cultures from mouse and human specimens that show functional neuromuscular connectivity post transplantation, holding potential for advancing regenerative therapies for neurointestinal diseases.

## Graphical abstract

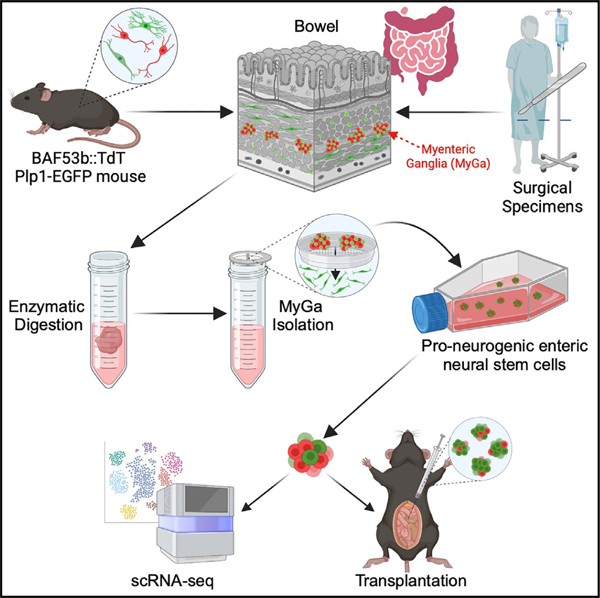

## INTRODUCTION

The enteric nervous system (ENS) is composed of an extensive interconnected network of enteric neurons and enteric glial cells (EGCs) that reside within the gut wall and regulate the numerous complex functions of the gastrointestinal (GI) tract, including absorption, secretion, barrier function, motility, and immunity.^1,2^ Acquired and congenital enteric neuropathies, including Hirschsprung disease, gastroparesis, esophageal achalasia, and others, result in severe morbidity due to severe GI dysmotility.^3^ Current treatment options for these conditions do not address the absence or loss of enteric neurons and EGCs underlying their pathophysiology but, rather, focus on symptom management or surgical removal of affected GI segments. Regenerative cell therapy is a promising treatment option that directly targets the fundamental problem of enteric neuropathies by replacing the missing neurons and glia with the potential to re-establish normal gut innervation and restore GI homeostasis. However, current cell therapy strategies that utilize ENS-derived progenitors have shown limited cell proliferation and neuronal differentiation, obstacles that need to be overcome to achieve clinical application. The goal of this study was to leverage what is known about postnatal enteric neurogenesis to develop strategies for the selective isolation and expansion of highly neurogenic progenitors and to test their ability to establish neuronal activity following *in vivo* transplantation.

Enteric neurons and EGCs arise from neural crest-derived cells, a progenitor cell population that migrates into and along the gut mesenchyme during embryologic development to form the ENS.^4,5^ In the postnatal GI tract, EGCs can rapidly proliferate in response to tissue injury,^6,7^ but enteric neurogenesis is slow following injury and essentially non-existent at steady state. Interestingly, a self-renewing population of enteric neuronal stem cells (ENSCs) can be isolated from the gut wall and exhibits high rates of neurogenesis *in vitro*. These ENSCs have been isolated from human gut tissue of all ages, ranging from neonates^8–10^ to the elderly,^11^ from both the small and large intestine^12^ and from both mucosal and full-thickness biopsies.^13^ ENSCs can be propagated in monolayer or free-floating neurosphere cultures and expanded exponentially.^9,11,14,15^ They can differentiate into both neurons and EGCs when transplanted into the embryonic^14,16^ and postnatal^14,17,18^ gut, but relatively low engraftment, proliferation, migration, and differentiation of transplanted cells could limit their success as a cell therapy.^4,18,19^ For example, while ENSCs have the ability to give rise to enteric neurons and neuronal subtypes following transplantation,^17^ the proportion of transplanted cells that differentiate into neurons is low.^14,17^ Furthermore, ENSCs are difficult to purify from other proliferating cell populations, and current isolation techniques include a significant proportion of non-progenitor cells.^20^ Cell therapies for ENS disorders would be vastly improved by increasing the number and proportion of ENSCs delivered to the recipient and by optimizing the capacity of the donor cells to generate neurons.

Accumulating evidence indicates that enteric neurogenesis arises from the transdifferentiation of a subset of EGCs in the postnatal environment.^6,21–23^ As the distinction between EGCs and ENSCs currently remains ambiguous *in vivo*, here, ENSCs refers to cultured EGCs with neurogenic potential. EGCs are widely distributed throughout the different layers of the gut, including within the submucosal and myenteric ganglia (MyGa) and along nerve fibers in the mucosa and intramuscular space.^24–26^ Recent *in silico* multiomic profiling of EGCs by our group identified that the intraganglionic EGCs residing in the MyGa may represent the major postnatal reservoir of EGCs with neurogenic potential *in vivo* as they remain epigenetically poised for neurogenesis by retaining open chromatin at genomic loci associated with enteric neuronal differentiation.^27^ Spatial restriction of neural progenitor populations is known to be present in the brain.^28,29^ The multiomics data referenced above point toward a similar spatial constraint in pro-neurogenic EGCs in the gut, although this has not previously been experimentally validated.

Here, we compare the neurogenic potential of ENS cells residing within the MyGa to those within the intramuscular space (intramuscular single cells [IM-SCs]). Current ENSC isolation methodologies that are commonly used generate single-cell suspensions containing predominantly IM-SCs. We hypothesize that many of the ENSCs residing within the MyGa are being discarded. We therefore established protocols to selectively isolate MyGa vs. IM-SCs to examine the neurogenic potential of each. Using lineage tracing models in mice, we show that MyGa favor enteric neurogenesis when compared to IM-SCs, leading to the expansion of a cell population markedly enriched for enteric neurons and their progenitors. MyGa-derived cells recapitulate appropriate enteric neuronal subpopulations and demonstrate neuronal functionality, including the generation of calcium transients following electrical field stimulation and neuromuscular connectivity following transplantation into the mouse intestine. Utilizing these insights, isolation procedures for the generation of ENSCs from human samples were developed and similarly yield a cell population with greater ENSC purity, high neurogenic potential, and neuronal functional properties following transplantation *in vivo*. These results confirm that highly neurogenic glia are spatially restricted to the MyGa and that the ability to selectively isolate and expand this population represents an important advance for the development of ENS cell therapy.

## RESULTS

### Intact MyGa can be isolated from the mouse intestine

**Figure 1.**
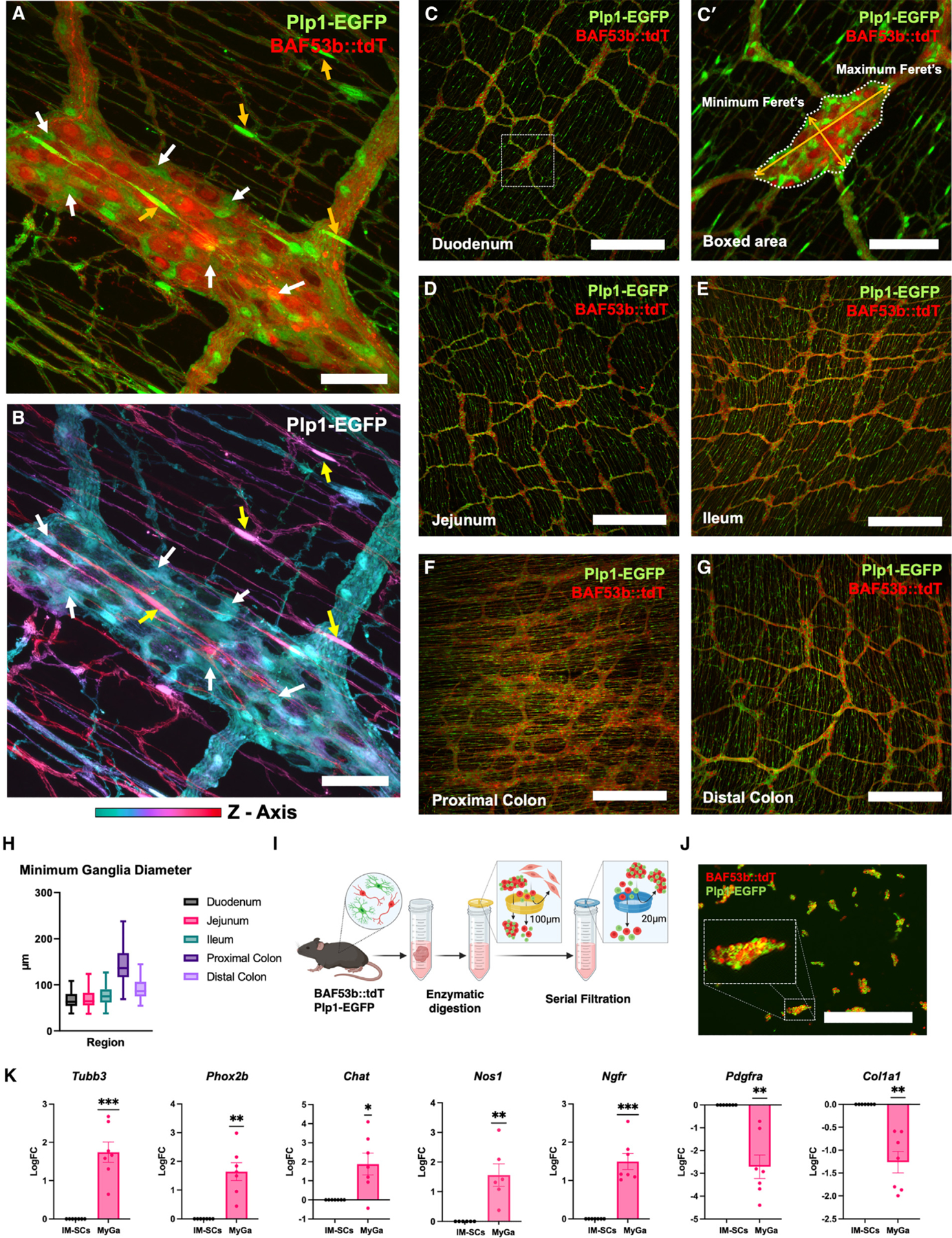
Intact MyGa can be isolated from the mouse intestine (A) Representative images of the myenteric ganglia (MyGa) of BAF53b::tdT; Plp1-EGFP mice, with Plp1-EGFP serving as a EGC marker and BAF53b::tdT as a neuronal marker. Scale bar: 50 μm. (B) Depth-coded projection of the z axis of Plp1-EGFP fluorescence, showing EGCs within the MyGa (white arrows) and EGCs in the IM space above (yellow arrows). Also see Video S1. Scale bar: 50 μm. (C–G) Representative images of the myenteric plexus from intestinal regions of BAF53b::tdT; Plp1-EGFP mice. Scale bars: 500 μm. (Cʹ) Minimum and maximum Feret’s diameters of a MyGa. Scale bar: 100 μm. (H) Quantification of the minimum Feret’s diameter of MyGa in intestinal regions. Boxplots are reported as mean ± 95% confidence interval (CI). *n* = 24 ganglia/segment. (I) Schematic overview of MyGa enrichment from the enzymatically digested bowel using counter filtration. (J) Representative image of the MyGa-enriched fraction after digestion from the gut of BAF53b::tdT; Plp1-EGFP mice. The inset shows a high-magnification view of a MyGa within the digested material. Scale bar: 500 μm. (K) Quantitative PCR of *Tubb3*, *Phox2b*, *Chat*, *Nos1*, *Ngfr*, *Pdgfra*, and *Col1a1* in digested single-cell suspensions and the MyGa-enriched fractions. One-sample t test to log fold change (LogFC) of 0, *p < 0.05, **p < 0.01, ***p < 0.001; *n* = 6–7 mice per group.

To visualize the myenteric plexus in mice, we utilized transgenic fluorescent reporter strains specifically marking enteric neurons and EGCs. For all experiments, mice were utilized after 3 months of age, when they had reached adulthood, to adequately reflect the postnatal environment.^30^ Enteric neurons were traced using BAF53b-Cre driver mice crossed with Rosa26-tdTomato (R26-tdT) reporter mice to generate BAF53b-Cre::R26-tdT (BAF53b:: tdT) mice. BAF53b-driven tdT fluorescence labels neuronal somata and their nerve fiber processes in the myenteric plexus.^31^ EGCs were visualized in mice with the glia-specific Plp1 promoter driving expression of EGFP. Double transgenics (BAF53b::tdT; Plp1-EGFP) allowed simultaneous visualization of neurons and glia in the myenteric plexus (Figure 1A). EGCs were observed adjacent to enteric neurons within the MyGa and throughout the IM region with bipolar processes projecting along enteric neuronal fibers (Figure 1B; Video S1). The sizes of the MyGa were quantified in BAF53b::tdT; Plp1-EGFP mice in different regions of the GI tract, including the duodenum, jejunum, ileum, proximal colon, and distal colon (Figures 1C–1G). The minimum Feret’s diameter was calculated, which describes the smallest dimension across the MyGa (Figure 1Cʹ). Calculations for minimum Feret’s diameters were similar across the intestinal segments, with the exception of the proximal colon, where MyGa were larger (Figure 1H).

Using these data, we calculated that 85% of the MyGa had a minimum diameter of 20–100 μm and developed processes to isolate the MyGa involving enzymatic digestion and particle size-based filtration. First the muscularis propria harboring the myenteric plexus was removed by manual microdissection and digested in an enzymatic cocktail to generate single-cell suspensions while retaining the enzymatically resistant MyGa as intact particles. Samples were filtered to collect the fraction <20 μm in size, comprising IM-SCs, and the fraction between 20 and 100 μm in size, containing enteric ganglia (Figure 1I). Visualization of BAF53b::tdT; Plp1-EGFP fluorescence confirmed that the latter fraction was enriched for enteric neurons and EGCs as compared to the IM-SC fraction (Figure 1J). The fraction containing material >100 μm was discarded. Validation by PCR showed that MyGa-enriched samples exhibited upregulation of neuronal (*Tubb3*, *Phox2b*, *Chat*, and *Nos1*) and EGC/ENSC (*Ngfr*) markers and downregulation of genes specific for mesenchymal cells (*Pdgfra* and *Col1a1*). These results confirm that the MyGa isolation strategy leads to a significant enrichment for ENS cells with reduced contamination by non-ENS cells as compared to the commonly utilized single-cell suspension (Figure 1K).

### MyGa-derived neurospheres have a high proportion of enteric neurons, glia, and progenitors

**Figure 2.**
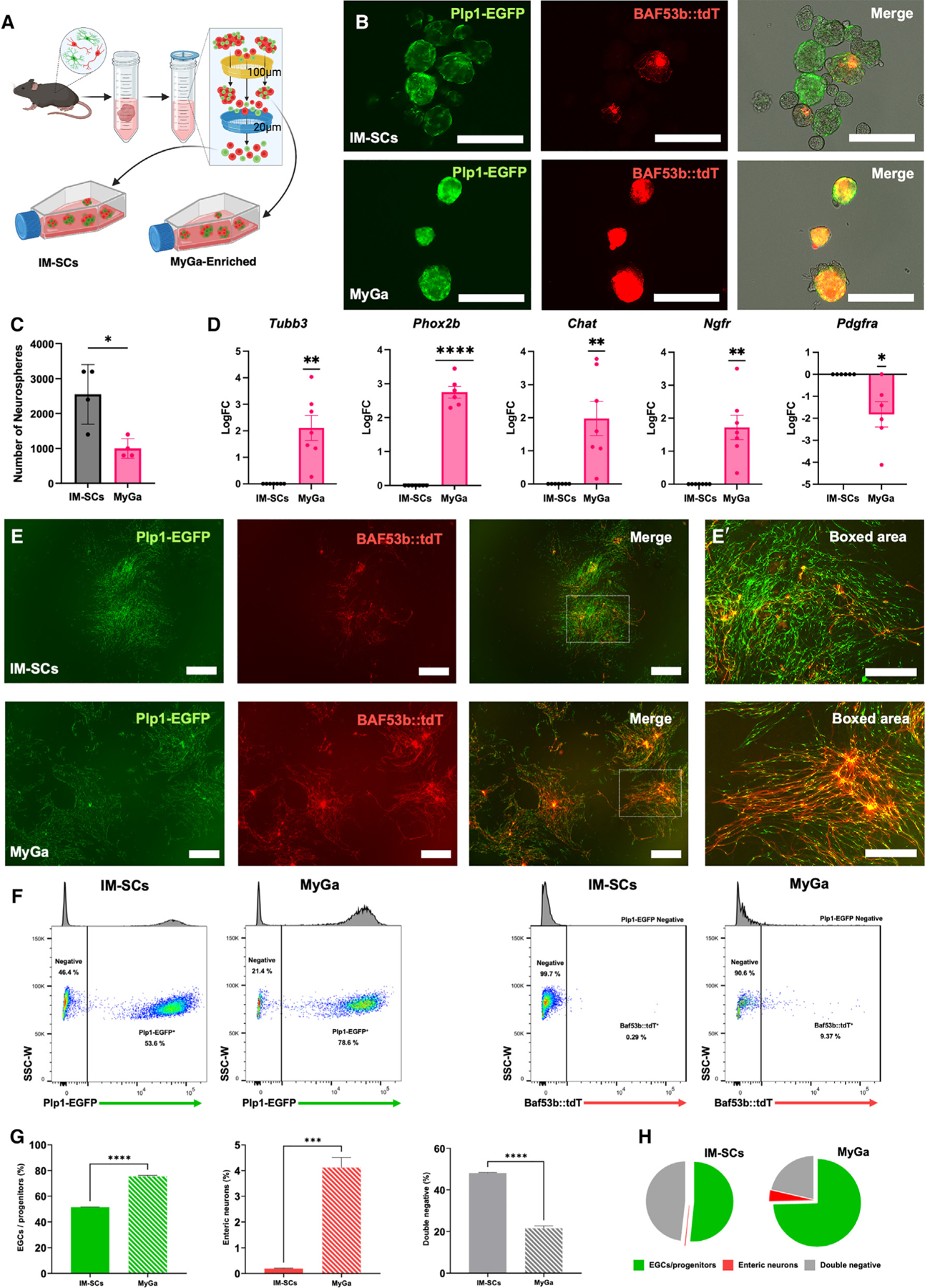
Neurospheres generated from the MyGa-enriched fraction have a higher purity of enteric neurons, EGCs, and progenitor cells (A) Generation of neurospheres from MyGa and the single-cell flowthrough. (B) Representative images of neurospheres generated from single-cell suspensions (IM-SCs) and the MyGa-enriched fractions from the small intestine of BAF53b::tdT; Plp1-EGFP mice. Scale bars: 250 μm. (C) Quantification of the number of neurospheres generated from single-cell suspensions and the MyGa-enriched fractions. Data are mean ± SEM. Unpaired t test, *p < 0.05, *n* = 3 mice per group. (D) Quantitative PCR of *Tubb3*, *Phox2b*, *Chat, Ngfr, Pdgfra,* and *Col1a1* in neurospheres generated from IM-SCs and the MyGa-enriched fractions. One-sample t test to LogFC of 0, *p < 0.05, **p < 0.01, *n* = 6–7 mice per group. (E and Eʹ) Cells from neurospheres after 1 week of migration, forming monolayers on fibronectin. Samples were originally generated from IM-SCs and the MyGa-enriched fractions as above. Scale bars: 1,000 μm (E) and 500 μm (Eʹ). (F) Flow cytometry data for EGCs/progenitors (Plp1-EGFP) and enteric neurons (BAF53b::tdT) derived from monolayer cultures. (G) Quantification of the number of EGCs/progenitors (Plp1-EGFP), enteric neurons (BAF53b::tdT), and double-negative cells from flow cytometry on monolayer cultures originally derived from IM-SCs and the MyGa-enriched fractions. Data are mean ± SEM. Unpaired t test, ***p < 0.001, ****p < 0.0001, *n* = 3 mice per group. (H) Pie chart representation of the proportions of EGCs/progenitors (Plp1-EGFP), enteric neurons (BAF53b::tdT), and double-negative cells from flow cytometry analysis.

To compare the neurogenic potential and cellular composition of IM-SCs versus MyGa preparations, serially filtered samples from the small intestine of BAF53b::tdT; Plp1-EGFP mice were cultured under free-floating conditions to form neurospheres (Figure 2A). Both preparations generated neurospheres suggestive of the presence of ENSCs. The expression of Plp1-EGFP and BAF53b::tdT was markedly elevated in MyGa-derived neurospheres (Figure 2B), although the number of spheres generated was lower (Figure 2C). As shown in Figure 1K, neuronal and EGC/ENSC markers were upregulated in the MyGa-derived neurospheres, while the mesenchymal marker *Pdgfra* was downregulated, suggesting that MyGa-derived neurospheres yielded a purer neuroglial and progenitor population *in vitro* (Figure 2D). To confirm this, neurospheres generated from MyGa and IM-SC preparations from BAF53b::tdT; Plp1-EGFP mice were cultured on fibronectin-coated plates, which facilitated cell migration into monolayers in both groups (Figure 2E–Eʹ). Following trypsinization into single-cell suspensions for flow cytometry (Figure 2F), MyGa-derived cultures were noted to be enriched for both Plp1-EGFP, representing ENSCs and EGCs, and BAF53b::tdT^+^ neurons (Figures 2G and 2H). Conversely, IM-SC cultures had greater proportions of contaminating non-ENS cells, as shown by their high expression of double-negative cells expressing neither neuronal nor glial markers (Figures 2G and 2H).

**Figure 3.**
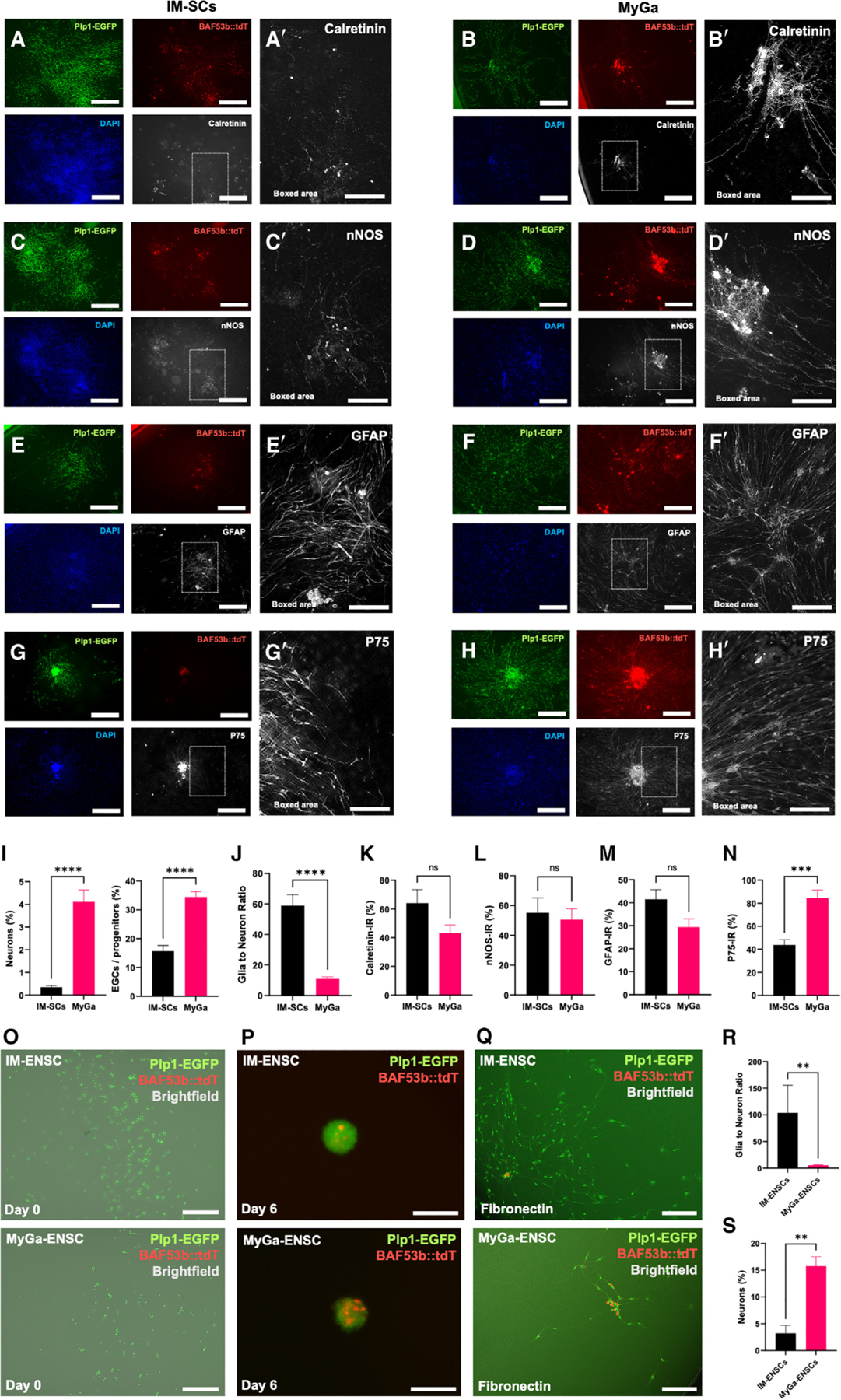
Neurospheres generated from MyGa retain neuronal subtypes important for the recapitulation of the ENS, and MyGa-derived ENSCs exhibit higher rates of enteric neurogenesis (A–Hʹ) Representative images of monolayer cultures originally derived from intramuscular single cells (IM-SCs) or the MyGa-enriched fractions immunohistochemically labeled for calretinin (A–Bbʹ), nNOS (C–Dʹ), GFAP (E–Fʹ), and P75 (G–Hʹ). Scale bars: 1,000 μm (A–H) and 250 μm (Aʹ–Hʹ). (I) Quantification of the percentage of EGCs/progenitors (Plp1-EGFP) and enteric neurons (BAF53b::tdT) in monolayer cultures, determined by fluorescent imaging. (J) EGC-to-neuron ratios in the same samples, determined by Plp1-EGFP and BAF53b::tdT fluorescence. Data are mean ± SEM. Unpaired t test, ****p < 0.0001, *n* = 19–20 independent cultures per group. (K–N) Quantification of the percentage of neurons (BAF53b::tdT) immunoreactive (IR) for calretinin (K) and nNOS (L) as well as the percentage of EGCs/progenitors (Plp1-GFP) IR for GFAP (M) and P75 (N). Data are mean ± SEM. Unpaired t test, ***p < 0.001, *n* = 5–7 independent cultures per group. (O) Plp1-EGFP cells immediately after purification by FACS of cultures derived from IM-SCs or MyGa-enriched fractions yielding IM-ENSCs and MyGa-ENSCs. Scale bars: 250 μm. (P) Expression of the neuronal marker BAF53b::tdT in neurospheres generated from IM-ENSCs and MyGa-ENSCs after 6 days in culture. Scale bars: 100 μm. (Q) IM-ENSC and MyGa-ENSC cultures after migration for 4 days on fibronectin. Scale bars: 200 μm. (R and S) EGC to neuron ratios (R) and the percentage of neurons to the total number of cells (S) in the same samples, determined by Plp1-EGFP and BAF53b::tdT fluorescence. Data are mean ± SEM. Mann-Whitney test, **p < 0.01, *n* = 6 independent cultures per group.

To determine how the neurosphere source affected their differentiation potential, neurospheres derived from IM-SCs and MyGa from BAF53b::tdT; Plp1-EGFP mouse intestine were cultured on fibronectin, and subpopulation markers were labeled by immunofluorescence (Figures 3A–3Hʹ). The proportion of tdT^+^ neurons and GFP^+^ EGCs was markedly higher in cultures derived from MyGa compared to IM-SCs (Figure 3I). Furthermore, the glia-to-neuron ratio was far greater in IM-SC samples, suggesting that MyGa give rise to a more neurogenic cell population (Figure 3J). Although the total number of enteric neurons was increased in MyGa-derived cultures, no differences were observed in the proportions of excitatory (calretinin) (Figures 3A, 3Bʹ, and 3K) versus inhibitory (neuronal nitric oxide synthase [nNOS]) neuron (Figures 3C, 3Dʹ, and 3L) subtypes between the two preparations when expressed as a percentage of BAF53b::tdT^+^ enteric neurons. This suggests that neurospheres generated from the MyGa and IM-SCs form similar neuronal subtypes important for recapitulating the ENS. There was no significant difference in the proportion of Plp1-EGFP cells co-expressing the EGC marker glial fibrillary acidic protein (GFAP) (Figures 3E, 3Fʹ, and 3M), while the proportion of Plp1-EGFP cells co-expressing the ENSC marker P75 was markedly elevated in the MyGa-derived fraction (Figures 3G, 3Hʹ, and 3N), suggesting the expansion of a pro-neurogenic ENSC population.

### MyGa-derived ENSCs exhibit higher rates of neurogenesis

Our data indicated that MyGa-derived neurospheres exhibit high levels of progenitor and neuronal markers, suggesting that these cells may contain a highly neurogenic population, consistent with our hypothesis that ENSCs are preferentially located within the MyGa.^27^ To experimentally validate the higher neurogenic potential of cells from the MyGa compared to IM-SCs, neurospheres were generated from BAF53b::tdT; Plp1-EGFP mice, cultured on fibronectin to form a monolayer, and trypsinized into single-cell suspensions for evaluation by fluorescence-activated cell sorting (FACS). In this experiment, cells were sorted for Plp1-EGFP, specifically selecting for a pure EGC/ENSC population and omitting differentiated enteric neurons (BAF53b::tdT). Cells expressing Plp1-EGFP were cultured under free floating conditions, and the absence of BAF53b::tdT expression was confirmed (Figure 3O). After 6 days in culture, both MyGa and IM-SCs formed neurospheres and contained neurons, as indicated by the presence of BAF53b::tdT fluorescence, confirming the neurogenic potential of Plp1-EGFP expressing ENSCs (Figure 3P). Neurospheres were then plated onto fibronectin-coated culture dishes to form monolayers and allow quantification of neuronal proportions (Figure 3Q). MyGa gave rise to populations with a lower glia-to-neuron ratio (Figure 3R) and a markedly greater proportion of BAF53b::tdT enteric neurons compared to IM-SCs (Figure 3S). The high rate of neurogenesis indicates that MyGa are indeed a reservoir for ENSCs with significant neurogenic potential.

### MyGa-derived neurospheres engraft, migrate, and differentiate into functional neurons following transplantation *in vivo*

**Figure 4.**
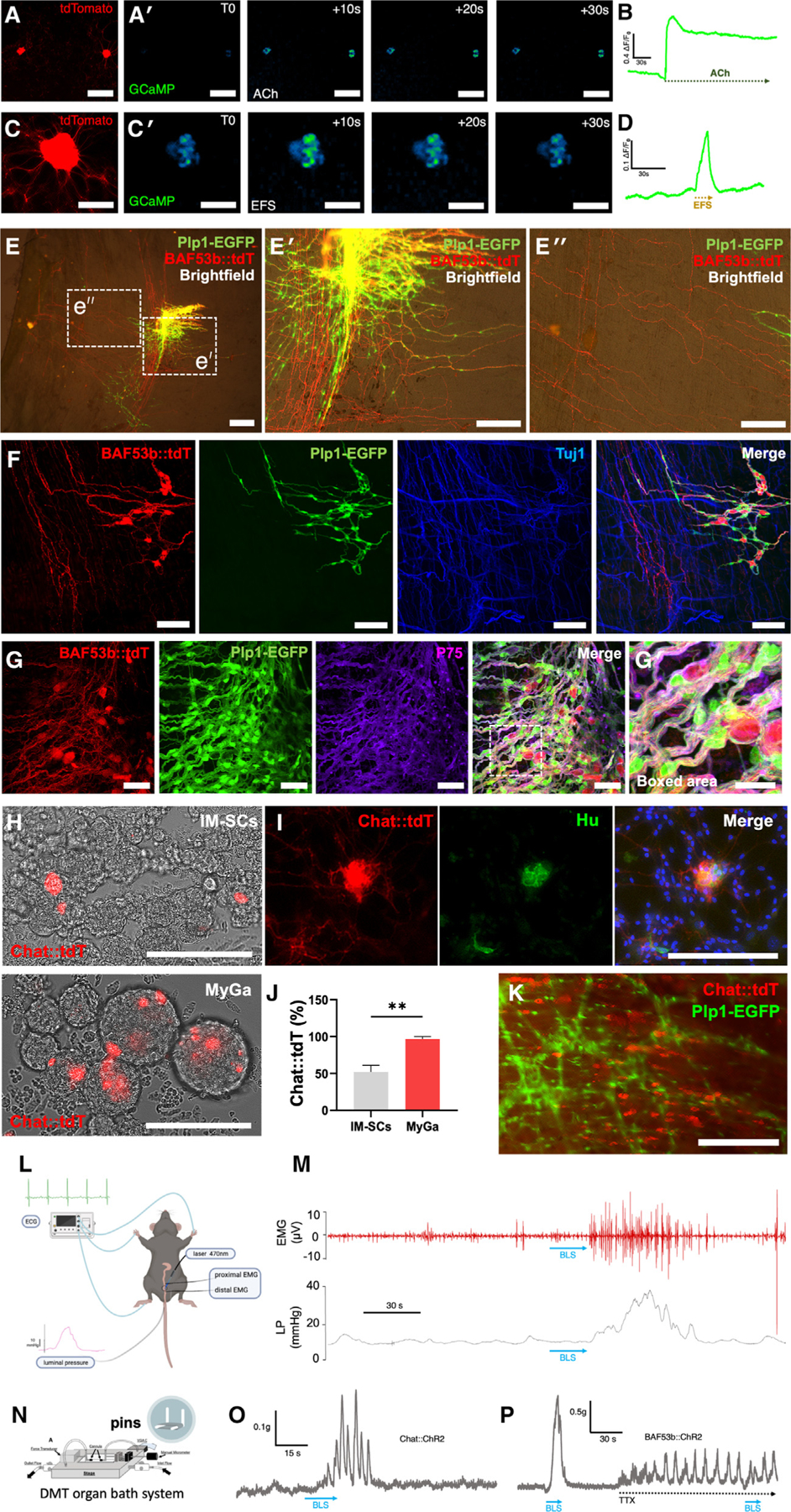
MyGa-derived cells generate calcium transients *in vitro* and show functional competency following transplantation to the intestine *in vivo* (A and Aʹ) Enteric neurons derived from MyGa cultures generated from BAF53b::tdT-GCaMP mice. Shown is expression of tdT in enteric neurons (A) and calcium transients in response to ACh (Aʹ). See also Video S2. Scale bars: 300 μm. (B) Representative traces of calcium transients (Δ*F*/*F*_0_) in response to ACh stimulation. (C and Cʹ) Enteric neurons derived from MyGa cultures from BAF53b::tdT-GCaMP mice as above with BAF53b::tdT expression (C) and calcium transients in response to electric field stimulation (EFS) (Cʹ). See also Video S3. Scale bars: 100 μm. (D) Representative traces of Δ*F*/*F*_0_ in response to EFS stimulation. (E) MyGa-derived neurospheres from BAF53b::tdT; Plp1-EGFP mice transplanted to the colonic muscularis propria of a colorless recipient. Scale bar: 500 μm. (Eʹ and Eʹʹ) High-magnification images of the cell transplantation site (Eʹ) and the extension of nerve fiber processes from the transplant (Eʹʹ). Scale bars: 250 μm. (F) Integration of transplanted BAF53b::tdT; Plp1-EGFP cells with the endogenous myenteric plexus of the recipient labeled by Tuj1. Scale bars: 100 μm. (G) BAF53b::tdT; Plp1-EGFP cell transplants expressing the progenitor marker P75. Scale bars: 50 μm. (Gʹ) Interconnected network formed between transplanted cells. Scale bars: 25 μm. (H) Neurospheres generated from IM-SCs and the MyGa-enriched fractions from Chat-tdT-ChR2 mice. Scale bars: 200 μm. (I) Chat-tdT expression in Hu IR enteric neurons in cultures of MyGa-derived cells. Scale bars: 200 μm. (J) Percentage of neurospheres from IM-SCs and MyGa-enriched fractions containing Chat-tdT neurons. Data are mean ± SEM. Unpaired t test, **p < 0.01, *n* = 3 independent cultures per group. (K) Chat-tdT cells from MyGa-derived neurospheres transplanted into the muscularis propria of a Plp1-EGFP recipient mouse. Scale bar: 200 μm. (L) Experimental setup for simultaneous recordings of luminal pressure and electromyography (EMG) *in vivo.* (M) *In vivo* EMG in the smooth muscle and intraluminal pressure of the colon in response to blue light stimulation (BLS) of transplanted Chat-tdT-ChR2 cells. (N) Experimental setup for colonic ring force contraction recordings *ex vivo*. (O) Force of contraction produced by colonic smooth muscle in response to BLS of transplanted Chat-tdT-ChR2 cells in *ex vivo* organ bath experiments. (P) Trace of the above with BLS of transplanted BAF53b::tdT-ChR2 cells and TTX inhibition of neural activity.

As the MyGa isolation process produces a cell population with increased neurogenesis and concomitant reduction in the contaminating mesenchymal population, MyGa may offer an improved therapeutic source of cells for ENS regeneration. To determine whether MyGa-derived cells exhibit neuronal properties, including responses to electrical field stimulation (EFS) and receptivity to the excitatory neurotransmitter acetylcholine (ACh), calcium imaging was performed on MyGa-derived cells cultured from BAF53b::tdT-GCaMP mice, in which neurons express a genetically encoded calcium indicator. Application of ACh to the culture medium elevated Ca^2+^ levels in the MyGa-derived cells (Figures 4A and 4B; Video S2), and EFS evoked Ca^2+^ transients consistent with neuronal action potentials (Figures 4C and 4D; Video S3), confirming the functionality of MyGa-derived neurons.

Next, we evaluated whether MyGa-derived neurospheres transplanted to the mouse intestine could successfully integrate with the endogenous ENS. MyGa-derived neurospheres were generated from BAF53b::tdT; Plp1-EGFP mice as described above, and 100 neurospheres were transplanted into the colonic wall of wild-type recipient mice. Four weeks after transplantation, expression of Plp1-EGFP and BAF53b::tdT from transplanted cells was observed, with extensive cell migration and projection of BAF53b::tdT nerve fibers within the intestinal smooth muscle (Figures 4E–Eʹʹ). In contrast, IM-SC-derived neurospheres did not engraft post transplantation at the same cell dose (MyGa 6/6 vs. IM-SC 0/4). tdT^+^ enteric neurons derived from MyGa co-expressed the neuronal marker TUBB3 and formed connections with host-derived TUBB3 immunoreactive nerve fibers as well as the MyGa of recipient mice, demonstrating integration with the endogenous ENS (Figure 4F). As seen in Figures 3H and 3N, we confirmed that transplanted MyGa-derived cells continued to express high levels of P75 4 weeks after transplantation (Figure 4G).

To demonstrate that donor neurons derived from MyGa were functionally competent in the host intestine, we conducted a series of transplantation experiments using optogenetic stimulation^32^ of donor cells and recorded evoked contractile responses in recipient intestinal smooth muscle. MyGa were isolated from the small intestine of Chat-Cre::R26-tdT-channelrhodopsin-2 (Chat::tdT-ChR2) mice, in which ChR2 is expressed by excitatory cholinergic neurons, which express choline acetyltransferase (ChAT). Neurospheres from MyGa were more likely to contain tdT expression compared to IM-SCs (Figures 4H–4J), and the neuronal specificity of the tdT expression was confirmed by co-labeling with the pan-neuronal antibody Hu (Figure 4I). Transplantation of Chat::tdT-ChR2 neurospheres from MyGa to Plp1-EGFP mouse recipients resulted in successful engraftment within the muscularis propria, with survival of transplanted cells for at least 4 weeks (Figure 4K). Recipient mice were anesthetized, and intraluminal colonic pressure was recorded in response to blue light stimulation (BLS), which optogenetically activates the transplanted Chat::tdT-ChR2 donor cells (Figure 4L). This was done in the presence of the nitric oxide donor sodium nitroprusside to inhibit spontaneous muscle activity. Stimulation of transplanted MyGa-derived cells by BLS evoked transient increases in luminal pressure indicative of their ability to cause contraction of the colonic smooth muscle, as demonstrated by *in vivo* electromyography (EMG) (Figure 4M). Recipient colons were then excised and dissected to obtain intestinal rings for organ bath experiments to measure the force of smooth muscle contractions (Figure 4N). In these preparations, BLS evoked robust smooth muscle contractions, confirming that the transplanted MyGa give rise to excitatory enteric neurons capable of making functional neuromuscular connections with the intestinal smooth muscle (Figure 4O). These experiments were replicated using MyGa derived from BAF53b::tdT-ChR2 (BAF53b::tdT-ChR2) mice, which express ChR2 on all neurons (Figure 4P). BLS of BAF53b::tdT-ChR2 expressing MyGaderived neurons in these preparations resulted in contraction of the smooth muscle, and this was inhibited by the addition of the sodium channel blocker tetrodotoxin (TTX), confirming that these contractions were mediated by transplantation-derived neurons (Figure 4P).

Together, these experiments confirm that MyGa-derived neurons possess electrical activity and responsiveness to neurotransmitters, integrate with the ENS after transplantation, and regulate smooth muscle contractility, thus demonstrating their capacity to differentiate into functional enteric neurons and their potential utility as a regenerative cell therapy.

### MyGa can be isolated from human intestinal muscularis propria

**Figure 5.**
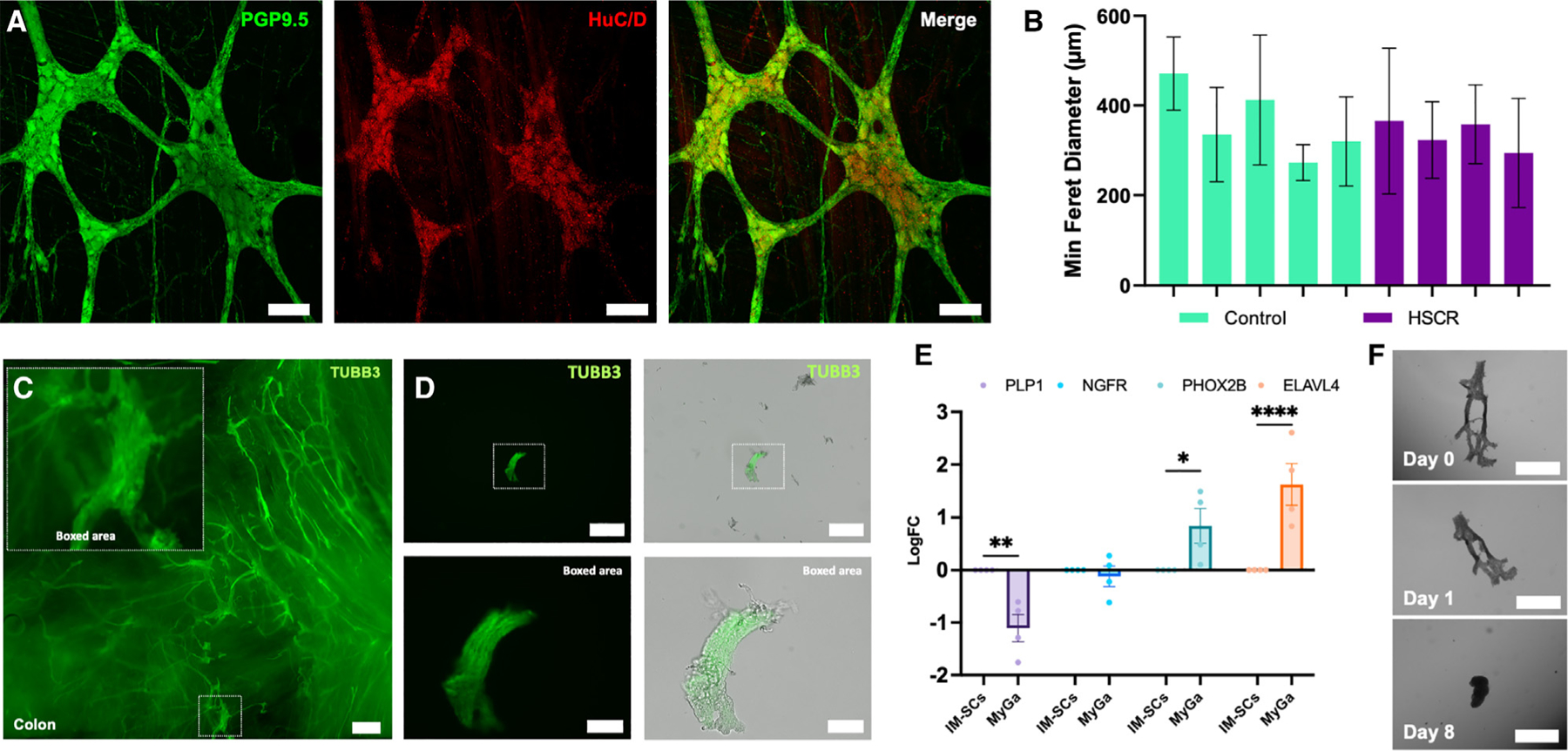
Intact MyGa can be isolated from the intestinal muscularis propria in resected human specimens (A) Representative images of the myenteric plexus in the muscularis propria from a resected colon immunohistochemically labeled with the pan-neuronal markers PGP9.5 and HuC/D. Scale bars: 100 μm. (B) Average minimum Feret’s diameter of MyGa from subjects with a normal ENS and the ganglionated segment of those with Hirschsprung disease (enteric neurocristopathy). Data are shown as mean ± 95% CI, *n* = 3–10 ganglia per sample (Data S1). (C) Immunohistochemical labeling of TUBB3 in whole-mount preparations of the muscularis propria and high-magnification image of the MyGa (boxed area). Scale bar: 1 μm. (D) Labeling of TUBB3 after enzymatic digestion of the muscularis propria. Scale bars: 500 μm. The boxed area shows higher magnification of a MyGa. Scale bars: 100 μm. (E) Quantitative PCR of *PLP1*, *NGFR*, *PHOX2B*, and *ELAVL4* in digested single-cell suspensions (IM-SCs) and the MyGa-enriched fractions of resected specimens. Data are shown as mean ± SEM. two-way ANOVA with Holm-Sidak post hoc test, *p < 0.05, **p < 0.01, ****p < 0.0001, *n* = 4 individuals per group. (F) Representative images of neurosphere formation from manually selected fragment of MyGa. Scale bars: 500 μm.

To examine the translational relevance of our findings, we next investigated (1) whether MyGa could be isolated from human intestinal samples and (2) whether, as in mice, these are similarly highly neurogenic and functionally effective following transplantation. First, the MyGa were visualized in human whole-mount preparations of the muscularis propria by immunohistochemistry for the pan-neuronal markers PGP9.5 and Hu (Figure 5A). The minimum Feret’s diameters were much larger in human specimens from both normal and Hirschsprung disease (HSCR) tissue than in mice, with minimum diameters ranging between 0.2 and 0.6 mm (Figure 5B). From these data, we predicted that the MyGa from human samples could be isolated using filter sets between 100 and 1,000 μm. To isolate the MyGa, full-thickness samples of intestine were obtained from clinically indicated surgical resections (Table S1). The muscularis propria was dissected as an intact sheet (Figure 5C), then minced and digested in a collagenase XI and dispase solution at 37°C for 4 h with gentle rotation throughout the digestion procedure before being serially passed through a filter set of 100 μm and then 1,000 μm. Samples were collected between 100 and 1 000 μm in size, with immunohistochemistry for the pan-neuronal marker TUBB3 revealing the successful isolation of intact human MyGa using these parameters (Figure 5D). For further validation, gene expression was compared between IM-SCs <100 μm and the 100–1,000 μm MyGa fraction. As in mice, the MyGa-enriched fraction contained significantly higher expression of the neuronal markers *ELAVL4* and *PHOX2B* (Figure 5E). To confirm whether neurospheres could be generated from human gut-derived MyGa, we first manually “picked” the MyGa under a dissection microscope, cultured them, and visualized them over 8 days. Time-lapse imaging of individual specimens indicated that the MyGa remodel over time to form neurospheres *in vitro* (Figure 5F). While hand-picking MyGa is too labor intensive for cell therapy applications, this confirms the neurogenic potential that these ganglia possess.

### Neurospheres generated from human MyGa are highly neurogenic

**Figure 6.**
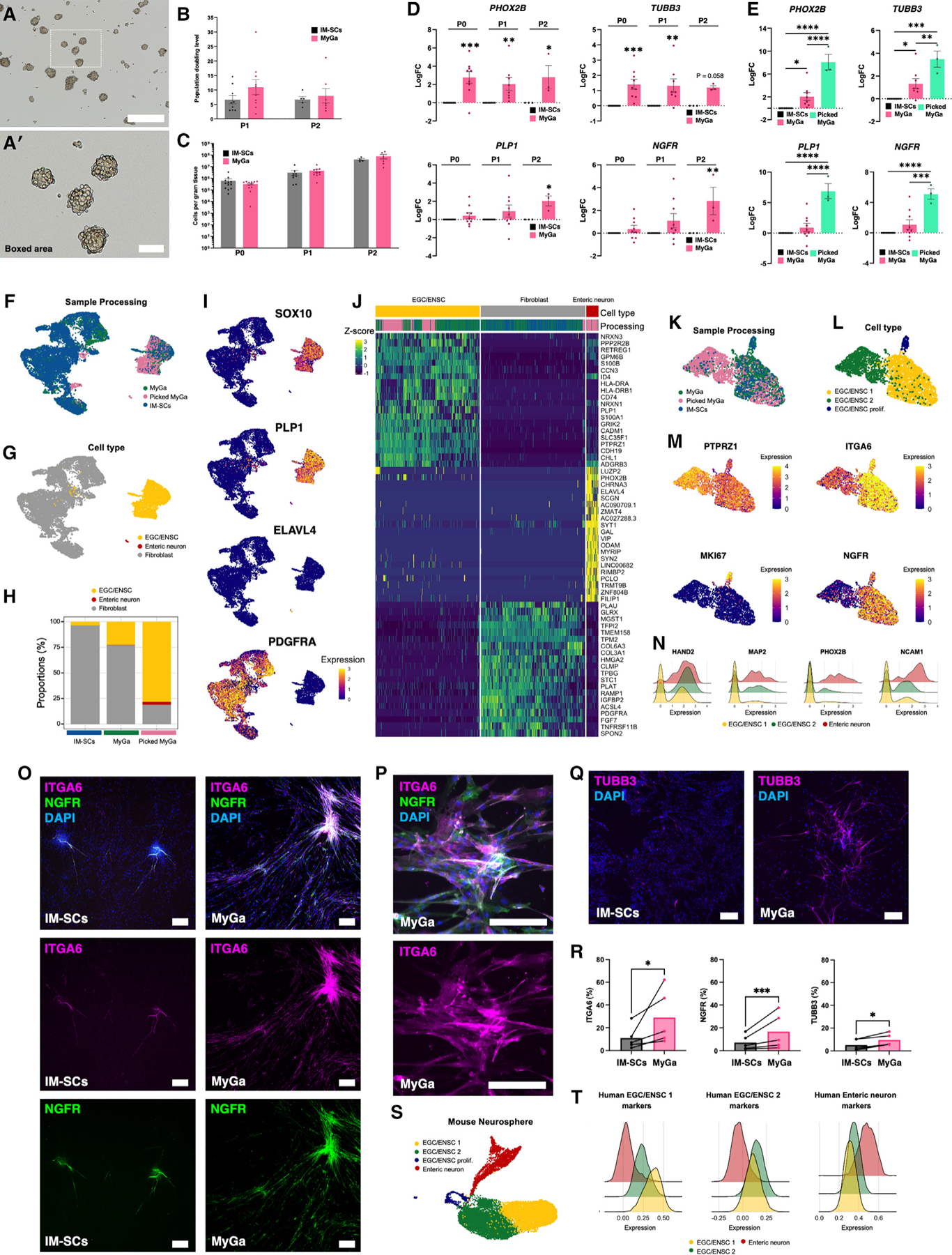
Neurospheres generated from human MyGa are highly neurogenic and contain ENSC subpopulations with distinct transcriptional profiles (A and Aʹ) Free-floating neurospheres generated from the MyGa-enriched fraction (counter filtered) of human specimens. Scale bars: 500 μm (A) and 100 μm (Aʹ). (B) Population doubling levels of cultures generated from IM-SCs and counter-filtered fragments enriched for MyGa at the first passage (P1) and second passage (P2). *n* = 5–10 individuals per group. (C) Total cell counts before cells were passaged (P0) and estimated total yield of cells from proliferation assays at the first and second passages normalized per gram of starting tissue. *n* = 4–12 individuals per group. (D) Quantitative PCR of *PHOX2B*, *TUBB3*, *PLP1*, and *NGFR* prior to passaging and at P1 and P2 in neurospheres generated from IM-SCs and the MyGa-enriched fractions of resected specimens. two-way ANOVA with Holm-Sidak post hoc test, *p < 0.05, **p < 0.01, ***p < 0.001, *n* = 3–9 individuals per group. (E) Quantitative PCR in neurospheres at P1 generated from IM-SCs, the MyGa-enriched fractions (MyGa), and pure hand-picked MyGa (picked MyGa). One-way ANOVA with Holm-Sidak post hoc test, *p < 0.05, **p < 0.01, ***p < 0.001, ****p < 0.0001, *n* = 3–9 individuals per group. (F and G) Uniform manifold approximation and projection (UMAP) representation of cells from cultures of IM-SCs, the MyGa-enriched fractions, and pure hand-picked MyGa (F) and unsupervised clustering (G) of cell populations. (H) Proportion of cell populations in cells generated from IM-SCs, the MyGa-enriched fractions, and pure hand-picked MyGa, defined by unsupervised clustering. (I) UMAP visualization of *bona fide* markers for EGC/ENSCs (*SOX10* and *PLP1*), enteric neurons (*ELAVL4*), and enteric mesenchymal cells (*PDGFRA*). (J) Heatmap visualization of the top 10 markers by LogFC (>60% of cells) for each cell population. Data are presented as *Z* scores. (K and L) UMAP representation of EGC/ENSC subpopulations from cultures of IM-SCs, the MyGa-enriched fractions, and pure hand-picked MyGa (K) and unsupervised clustering (L) of populations. (M) UMAP visualization of gene expression markers for human EGC/ENSCs and their subpopulations. (N) Ridge plot of the expression of neuronal markers and pro-neurogenic factors in EGC/ENSCs and enteric neurons. (O–Q) Representative images of immunocytochemical labeling of ITGA6, NGFR (O and P), and TUBB3 (Q) in monolayer cultures. Scale bars, 200 μm. (R) Quantification of the proportion of cells expressing ITGA6, NGFR, and TUBB3. *n* = 5 subjects per group, ratio paired t test, *p < 0.05, ***p < 0.001, *n* = 5 individuals per group. (S) Clustering of cells from cultures of mouse neurospheres. (T) Expression module scores for human enteric neuron and EGC/ENSC 1 and 2 population markers in mouse neurosphere cell populations.

To determine whether isolation of human MyGa utilizing the filtration method could be leveraged for ENS regeneration, we compared the properties of neurospheres generated from IM-SCs traditionally used to culture enteric neurospheres (<100 μm) to those derived from the MyGa-enriched fraction (100–1,000 μm). MyGa-derived neurospheres formed under appropriate culture conditions (Figures 6A and 6Aʹ) were cultured on fibronectin to form a monolayer before trypsinization to yield single-cell suspensions for quantification of cell yield. No difference was observed in the proliferation capacity of cells isolated from the MyGa versus IM-SC fractions during the first or second passage (Figure 6B). Furthermore, unlike in mice, the MyGa enrichment process did not reduce the overall cell yield before the cells were passaged (passage 0 [P0]) or the anticipated cell yields per gram of tissue determined by population doubling level assays at P1 and P2 (Figure 6C). To determine the effects of MyGa enrichment on the properties of neurospheres, expression of neuronal markers (*PHOX2B* and *TUBB3*) and EGC/ENSC markers (*PLP1* and *NGFR*) was determined across the three passages. The pro-neuronal markers *PHOX2B* and *TUBB3* were elevated in MyGa-derived neurospheres at P0 and P1, but no statistical differences in *PLP1* and *NGFR* were observed until P2 (Figure 6D). To validate that the enrichment of ENSCs in the MyGa fraction was responsible for these results, additional experiments were performed comparing neurospheres derived from hand-picked MyGa to those from the MyGa-enriched and IM-SC preparations at the first passage. Neurospheres isolated from hand-picked MyGa demonstrated the highest levels of *PHOX2B*, *TUBB3*, *PLP1*, and *NGFR*. As these samples contain the purest source of MyGa, this confirms that the MyGa are the major source of ENSCs in the human intestine and likely explain the elevated expression of these genes in the MyGa-enriched samples (Figure 6E).

To further resolve the cell populations, single-cell RNA sequencing (scRNA-seq) was performed on cultures derived from the commonly utilized IM-SC suspensions, MyGa-enriched fractions, and hand-picked MyGa (Figure 6F). In the combined dataset, we observed distinct clusters of fibroblasts, EGC/ENSCs, and a small population of neurons (Figure 6G). As seen in our PCR results, cultures derived from hand-picked MyGa exhibited the highest purity of EGC/ENSCs, and those derived from the MyGa-enriched fraction improved ENSC purity 6.1-fold compared to IM-SC preparations (Figure 6H). These findings highlight the important observation that ENS progenitors are spatially restricted to the MyGa. Cell populations were validated by the expression of *bona fide* markers for EGCs (*SOX10*, *S100B*, and *PLP1*), ENSCs (*SOX2*, *ERBB3*, *FOXD3*, and *GFRA1*),^33^ enteric neurons (*ELAVL4*, *MAP2*, *PHOX2B*, and *L1CAM*), and intestinal fibroblasts (*PDGFRA*) (Figures 6I and S1A). Previously proposed markers of human muscularis EGCs^34^ were also expressed by EGC/ENSCs, further validating their identity (Figure S1A). The top 10 markers by fold expression in these populations (>60% of cells) included known markers of neurons (*VIP* and *GAL*) and intestinal fibroblasts (*COL6A3*), while the EGC/ENSC population exhibited high expression of *PLP1*, the marker utilized in this study to isolate EGC/ENSCs from mice (Figure 6J).

The heterogeneity of EGC/ENSCs was further explored, and 3 main subpopulations were identified: EGC/ENSCs actively undergoing cell proliferation and two transcriptionally distinct EGC/ENSC subsets (Figures 6K and 6L). The 3 subpopulations of EGC/ENSCs shared markers such as *PTPRZ1* and *ITGA6*, which have been detected in mouse embryonic EGC/ENSCs^35^ (Figure 6M)*. MKI67* was confirmed in the proliferating EGC/ENSC cluster, while the traditionally utilized ENSC marker *NGFR* was not a reliable pan-ENSC marker in humans but, rather, was predominately expressed in the EGC/ENSC-1 subpopulation (Figure 6M). To evaluate the pro-neurogenic properties of EGC/ENSCs, differential expression of enteric neuronal markers was assessed between the two EGC/ENSC subpopulations. Only 23 of 581 neuronal markers were upregulated in EGC/ENSC-1 compared to 133 of 581 in EGC/ENSC-2, including the established pro-neurogenic genes *HAND2* and *PHOX2B* and neuronal markers *MAP2* and *NCAM1* (Figure 6N).

To validate EGC/ENSC markers identified by scRNA-seq in human samples, cells were labeled via immunocytochemistry. The EGC/ENSC 1 marker NGFR labeled a subset of the predicted pan-EGC/ENSC marker ITGA6, as anticipated (Figures 6O and 6P). Quantification of ITGA6, NGFR, and TUBB3 indicated that ITGA6 was the most abundant EGC/ENSC marker and, furthermore, that MyGa enrichment improves the purity of cell cultures for EGC/ENSCs and enteric neurons (Figures 6Q and 6R).

To determine whether these EGC/ENSCs subpopulations were specific to the human gut, their possible equivalencies were explored in mouse neurospheres (Figure 6S). Scoring of human enteric neurons and EGC/ENSC 1 and 2 subpopulation markers for homologous genes in the mouse revealed strong correlations between mouse and human enteric neurons and both EGC/ENSC subpopulations (Figure 6T). Notably, the predicted mouse EGC/ENSC 2 population scored higher for neuronal genes and was situated near the neuroblast/neuronal cluster, suggesting that both species contain a highly neurogenic EGC/ENSC population (Figures 6S and 6T). Shared characteristics of EGC/ENSC 1 in mice and humans include higher expression of *NGFR* and *RUNX2* (nerve regeneration), whereas EGC/ENSC 2 share higher expression of *SLC35F1* (neurodevelopment) and *PTGDS* (neuromodulator) (Figure S1B). Markers of human EGC/ENSC 1 and 2 populations resembled two polarized EGC populations in the Drokhylansky et al. human intestinal atlas, suggesting that traits of EGC populations could remain *in vitro* (Figures S1C–S1E). Transcriptional markers for mouse intraganglionic EGCs were evaluated in the datasets of cultured EGC/ENSCs from mouse and human samples (Figure S1F). The EGC/ENSC 2 population exhibited higher expression of intraganglionic EGCs in both species (mouse, 9 of 11 genes; human, 10 of 11 genes), lending support to the possibility that EGC/ENSC 2 is an equivalent population in mice and humans that shares characteristics with MyGa EGCs (Figure S1F).

### Human MyGa ENSCs are primed for regenerative cell therapy

**Figure 7.**
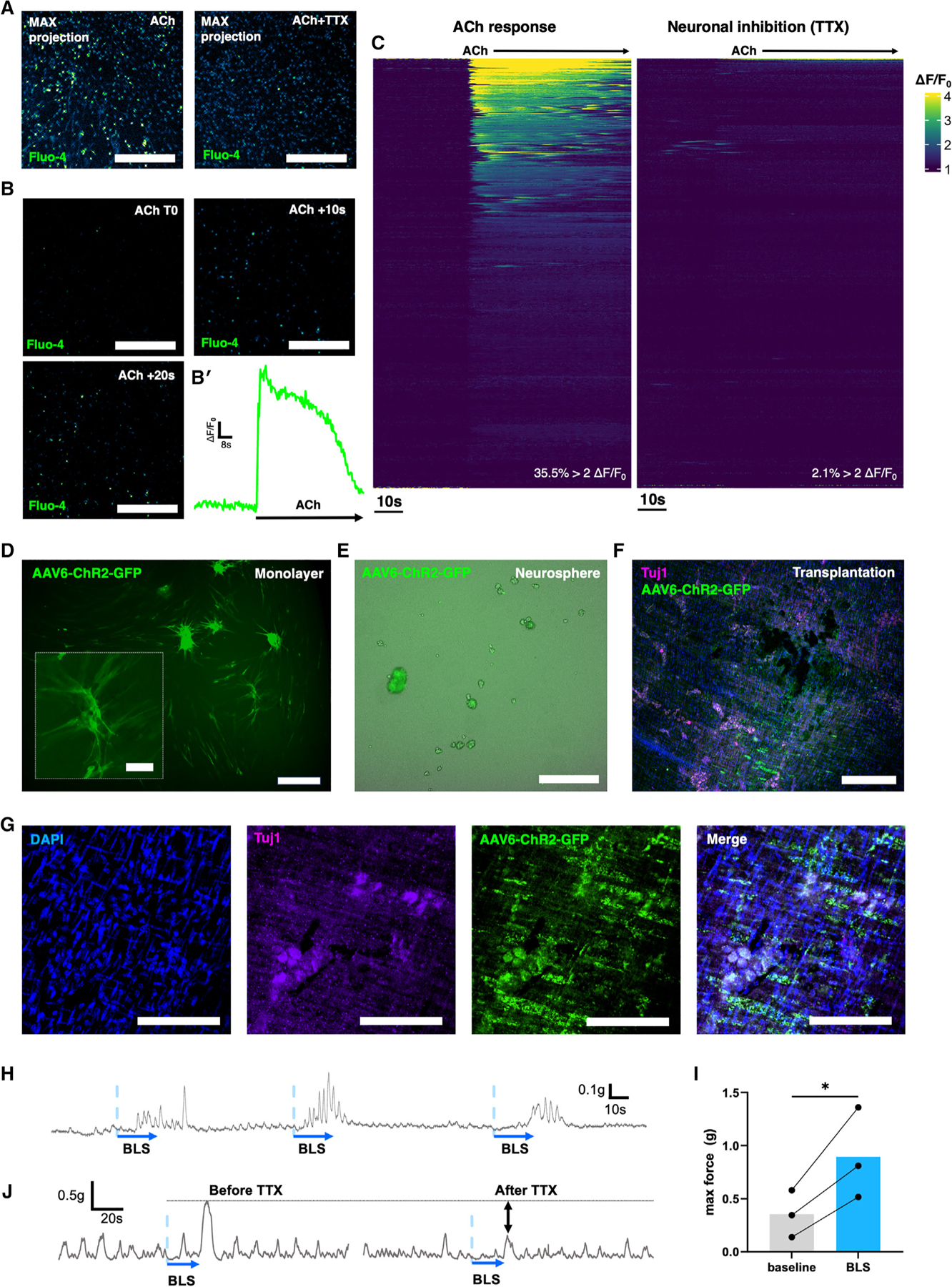
Human MyGa-derived cells demonstrate functional competency and have utility for cell therapy applications (A) Maximum-intensity projections of calcium responses over time using the Fluo-4 calcium indicator in human MyGa-derived cultures stimulated with ACh or with ACh stimulation following pretreatment with tetrodotoxin (TTX). Scale bars: 500 μm. See also Video S4. (B) Still images of calcium transients using the Fluo-4 calcium indicator following the application of ACh (T0) after 10 and 20 s. Scale bars: 500 μm. (Bʹ) Representative trace of a calcium response (Δ*F*/*F*_0_) to ACh in an individual cell. (C) Heatmap of Δ*F*/*F*_0_, with the y axis representing individual cells and the x axis representing time. (D) Expression of the GFP reporter after transduction of MyGa-derived cells with AAV6-ChR2-GFP in monolayer cultures on fibronectin. Scale bar: 500 μm. Inset scale bar: 100 μm. (E) Expression of GFP in AAV6-ChR2-GFP-transduced cells after reformation of neurospheres under free floating conditions. Scale bar: 500 μm. (F) Representative image of transplanted MyGa-derived cells to the muscularis propria of NOD-SCID IL-2Rgamma^null^ (NSG) mice after 3 weeks of engraftment. Samples were labeled for the neuronal marker Tuj1 and stained with DAPI. Scale bar: 200 μm. (G) High-magnification images of transplanted cells defined by GFP expression and overlapping expression of Tuj1. Scale bar: 100 μm. (H) Force of contraction produced by colonic smooth muscle in response to BLS of transplanted cells expressing AAV6-ChR2-GFP in *ex vivo* organ bath experiments. (I) Quantification of contractile force in colonic smooth muscle in response to BLS compared to baseline measurements in the same sample. Paired t test, *p < 0.05, *n* = 3 mice per group. (J) Representative traces of smooth muscle contractions in the presence of TTX.

To determine whether human ENSCs derived from the MyGacould be utilized as a cell therapy, we assessed (1) their ability to evoke calcium transients (effector function) and (2) their ability to induce colonic smooth muscle contractions (effector function). Calcium responses to ACh were determined using time--lapse imaging of monolayer cultures containing MyGa using the cell-permeable calcium indicator Fluo-4 (Figures 7A–7C). To determine whether ACh-induced transients were neuronally mediated, cultures were preincubated with TTX to inhibit neuronal activity. Maximum intensity projections across time-lapse imaging show a higher number of cells responding to ACh compared to those in TTX conditions, suggesting that calcium responses are consistent with neuronal activity (Figure 7A; Video S4). Typically, cells remained inactive until the application of ACh, which induced a sharp rise in intracellular Ca^2+^ within seconds, followed by a steady decline in Ca^2+^ levels over approximately 1 min (Figures 7B and 7Bʹ). Evaluation of intracellular Ca^2+^ responses to ACh (*n* = 226 cells) and to ACh in the presence of TTX (*n* = 304 cells) indicated that 35.5% of MyGa-derived neurons responded to ACh, as determined by a doubling in fluorescence levels and only 2.1% responders in the presence of TTX (Figure 7C).

To evaluate the ability of human MyGa-derived neurons to regulate smooth muscle activity, ENSCs were transduced with an adeno-associated virus (AAV) vector expressing ChR2 and a GFP reporter (Figure 7D). These cells were cultured to form neurospheres (Figure 7E) and then transplanted into the colonic muscularis propria of non-obese diabetic (NOD)-severe combined immunodeficiency (SCID) interleukin-2R (IL-2R)gamma^null^ (NSG) immunocompromised mice. After 3 weeks, transplants were assessed for the presence of GFP expression, indicative of engraftment by successfully transduced human cells (Figures 7F and 7G). Colonic rings were collected from the transplantation site for organ bath experiments to measure smooth muscle contractility by optogenetics using BLS. Representative traces show a consistent pattern of smooth muscle contractions in response to BLS of the transplanted region (Figure 7H). The peak force was quantified and shows that BLS of transplanted human MyGa-derived cells enhances smooth muscle contractions, indicating that they develop functional neuromuscular connectivity after transplantation (Figure 7I). Elevations in peak force in response to BLS were negated in the presence of TTX, confirming that these effects were neurally mediated (Figure 7J).

## DISCUSSION

In this study, we develop protocols that allow for EGCs residing within the MyGa in both mice and humans to be isolated and cultured independently. Using a lineage tracing mouse model, we demonstrate that the MyGa-EGC/ENSCs are more neurogenic than the EGC/ENSCs inhabiting the IM space, leading to the expansion of a purer cell population that is markedly enriched for enteric neurons and their progenitors with reduced contamination by non-ENS cells. Importantly, MyGa-derived neurons recapitulate appropriate neurochemical subpopulations, including excitatory (calretinin) and inhibitory (nNOS) neurons, and demonstrate functionality by generating calcium transients following EFS and by exhibiting direct control over intestinal muscle contractility following transplantation and optogenetic activation. Similar experiments were conducted on human samples, yielding a population with higher ENSC purity that favored neuronal differentiation and demonstrated functional neuronal properties following *in vivo* transplantation to mice.

This study provides an extensive single-cell transcriptome dataset of human-derived enteric neurospheres. Our sequencing data not only validate the efficacy of our isolation process, confirming both the enrichment of ENS cells from MyGa as well as their associated enhanced neurogenic potential, but also offer insights into potential subpopulations of EGC/ENSCs and their transcriptional markers. We categorize two subpopulations of EGC/ENSCs that exhibit distinct profiles across samples and find the ENSC-2 population to be enriched in MyGa. Interestingly, the ENSC-2 subpopulation exhibited higher expression of pro-neurogenic and neuronal markers, including *MAP2*, consistent with our *in vitro* findings that MyGa-derived cells have increased neurogenic differentiation potential. Furthermore, using a viral vector expressing ChR2 and a GFP reporter, we demonstrate successful engraftment of human-derived enteric neurons into an immunocompromised mouse host and subsequent effective smooth muscle contraction following BLS. These results provide pivotal evidence of functional human gut-derived cells post transplantation, which is a critical step for the future of regenerative therapy.

Prior evidence suggests that postnatal enteric neurogenesis arises from the transdifferentiation of a subset of EGCs.^6,21–23,27,36^ Constitutive adult enteric neurogenesis does not occur under steady-date conditions *in vivo* but, rather, only after specific perturbations to the gut. Using a lineage marking system, one study found a subset of EGCs to differentiate into enteric neurons 3 months after the direct chemical ablation of the myenteric plexus.^21^ Another group demonstrated that chemical colitis triggered robust neurogenesis by Sox2^+^ and Plp1^+^ EGCs^22^ and that this response was dependent on serotonin signaling.^23^ Although it is now well recognized that EGCs can become enteric neurons under the right conditions, the exact nature and location of this neurogenic glial subtype is unknown. EGCs are broadly distributed throughout the different layers of the intestinal wall, including within the submucosal and MyGa and in close proximity to nerve fibers extending into the mucosa and IM space.^24,25^ Four types of EGCs have been described previously based on morphology and location, where type I EGCs are astrocyte like and found within the ganglia, type II EGCs are fibrous in appearance and located within interganglionic connectives, type III EGCs are at the level of the myenteric plexus but located outside the ganglia in close association with neuronal fibers, and type IV EGCs have a bipolar morphology and are located within the circular and longitudinal smooth muscle along nerve fibers.^24^ In the brain, specific “neurogenic zones” have been identified, specifically the subgranular zone of the hippocampus and the subventricular zone of the lateral ventricles, indicating that neural progenitor populations in the brain are spatially restricted.^28,29^ Whether a similar spatial restriction applies to enteric neural progenitor populations has not been determined. Recent multiomics profiling of EGCs by our group identified that the EGCs residing within the MyGa may represent the major postnatal reservoir of multipotent ENSCs, as they retain open chromatin at loci associated with neuronal differentiation, remaining epigenetically poised for neurogenesis.^27^ Our work validates these prior *in silico* findings, specifically revealing that the MyGa (containing predominantly type I EGCs) are significantly more neurogenic than IM-SCs residing with the IM space (containing predominantly type IV EGCs), and suggests a spatial restriction to enteric neural progenitor populations similar to that identified previously in the brain.

The specific isolation, propagation, and transplantation of MyGa-derived cells may offer significant therapeutic benefits for the treatment of ENS disorders in the form of cell therapy. Regenerative therapies represent a promising strategy to replace the missing neurons and glia by restoring function in specific ENS disorders,^20^ including gastroparesis^37^ and Hirschsprung disease.^10,38,39^ ENSCs can differentiate into enteric neurons and EGCs following transplantation into both the embryonic^14,16^ and postnatal^14,17,18^ intestine and are capable of specifically differentiating into neurons of the appropriate neurochemical phenotype.^17^ Although cell therapy offers promise for the treatment of neurointestinal diseases, translation has been hampered by the relatively low fraction of cells that differentiate into enteric neurons^14,17^ and the challenge of separating ENSCs from other cell types, resulting in a significant population of non-ENS progenitor cells. For these reasons, there has been a relatively limited ability to recapitulate a functional ENS following the engraftment of ENSCs, restricting the overall success of cell therapy.^4,18,19^ Current isolation techniques for ENSCs utilize small filters that predominantly isolate IM-SCs and discard many of the neurogenic EGCs residing within the MyGa. Using our proposed isolation process that enriches for MyGa, we simultaneously increase the proportion of ENSCs and decrease the proportion of non-progenitor cells that are delivered to the recipient while also enhancing the neurogenic differentiation potential of the transplanted ENSCs. This optimization of cell isolation strategies will offer significant value toward improving regenerative cell therapies for the treatment of ENS diseases.

### Limitations of the study

While this study offers promising insights into the isolation, characterization, and therapeutic potential of MyGa-derived ENSCs, there are several limitations that should be acknowledged. First, this study examined only the short-term outcomes of MyGa-derived neurosphere transplantation. The long-term survival, integration into the host ENS, and functional capacity of MyGa-derived ENSCs need to be ascertained. Second, only wild-type mice were utilized as recipients in *in vivo* transplantation experiments, and further validation in mouse models of enteric neuropathy is warranted. Further studies evaluating the efficacy and utility of transplanting human MyGa-derived ENSCs onto *ex vivo* human tissue should also be performed. Last, there are approximately 20 functional classes of enteric neurons with distinct neurochemical coding, cellular morphology, or target projections,^1,2^ and it is currently unclear to what extent the neurons generated from ENSCs can recapitulate these complex resident neuron populations and circuitry. While our study includes scRNA-seq data on human ENS cells, we have insufficient numbers of neurons to identify the presence of specific subpopulations with confidence. Furthermore, the effects of gender were not investigated in detail. The limitations highlighted are important areas for future investigation.

## RESOURCE AVAILABILITY

### Lead contact

Requests for further information, resources, and reagents should be directed to and will be fulfilled by the lead contact, Rhian Stavely (rstavely@mgh.harvard.edu).

### Materials availability

This study did not generate new unique reagents.

### Data and code availability

- All data used in this manuscript have been made available as Data S1 and S2. Raw single-cell RNA sequencing data are available upon request from the lead contact. Gene count files have been deposited at Dataverse and are publicly available as of the date of publication. The DOI is listed in the key resources table.
- All original code has been deposited at Zenodo and is publicly available as of the date of publication. The DOI is listed in the key resources table.
- Any additional information required to reanalyze the data reported in this paper is available from the lead contact upon request.

## STAR★METHODS

### EXPERIMENTAL MODEL AND SUBJECT DETAILS

#### Ethical statement

Animal experimentation was performed according to experimental protocols approved by the Institutional Animal Care and Use Committees IACUC (2009N000239) of Massachusetts General Hospital. This study was approved by the human research ethics committees of Massachusetts General Hospital (IRB protocol #2010P00669), The Royal Children’s Hospital, and Monash University (HREC 38262). Written consent was collected for all subjects prior to participation in this study.

#### Mice

Details for all mice utilized in this study are provided in the key resources table. Mice were purchased from Jackson laboratory (Bar Harbor, ME) with the exception of Plp1-EGFP mice which were kindly donated by Wendy Macklin PhD, University of Colorado.^29^ All mice were housed at the Center for Comparative Medicine animal facility at Massachusetts General Hospital under specific-pathogen-free conditions. All experiments were approved by the Massachusetts General Hospital Institutional Animal Care and Use Committee IACUC (2009N000239). Rodents were housed in Allentown Inc rectangular caging (160 cages per individually ventilated cage racks; which uses blower at 60 air changes per hour) under a 12h:12h light:dark cycle from 7 a.m.–7 p.m. Bedding consisted of Hardwood Sanichip; with Carefresh nesting material and mice had access to Prolab Isopro RMH 3000 chow mix (ScottPharma) *ad libitum*. For all experiments, both male and female mice were utilized after 3 months of age when they had reached adulthood.^30^

#### Human specimens

Specimens of ganglionated Hirschsprung disease (HSCR) colon were taken from patients (4–21 months of age) during pull-through surgery at the Royal Children’s Hospital, Melbourne, Australia. Control samples were obtained during closure of stoma surgery for anorectal malformation (ARM) from patients ranging 9–20 months of age. ARM tissue has normal intrinsic enteric innervation and hence is considered as a ‘healthy’ control in this study. Excess colonic or ileal intestinal samples resected as part of required patient care at Massachusetts General Hospital were collected from subjects between 2 months and 61 years old (Table S1).

### METHOD DETAILS

#### Quantification of ganglia sizes in mouse samples

To simultaneously visualize enteric neurons and EGCs in the intestine, dual reporter BAF53b-CreR26-tdT; Plp1-EGFP mice (BAF53btdT; Plp1-EGFP) were bred as we previously reported.^27,42^ Mice were euthanized by CO_2_ asphyxiation and the small and large bowel were collected to prepare wholemount preparations by opening the intestinal lumen by cutting along the mesenteric border, pinning the tissue onto silicone-lined petri-dishes and removing the mucosa and submucosa under a dissection microscope prior to fixation in 4% PFA for 4 h. The remaining muscularis propria containing the myenteric plexus were then imaged using a Keyence BZX-700 All-In-One Microscopy system (Keyence America Itasca). Morphological characterization of the ganglia was performed in 10 random ganglia captured within a total field of view of 2 mm^2^ per tissue segment and per mouse. Individual ganglia containing neuronal soma were manually traced using Fiji open-source software (ImageJ v1.54f^43^) and Feret’s diameters for each ganglia were calculated using the in-built measurements function in Fiji.

#### Quantification of ganglia sizes in human tissue samples

Tissue specimens from HSCR and ARM subjects were collected following surgical removal and transported in cold phosphate buffered saline (PBS, 0.1M, pH7.4) for processing. Mesentery and associated fat were removed, then tissues were placed in PBS containing nicardipine (10 μM) to limit contractions. Tissue was stretched to form a flat sheet and pinned mucosa downwards on a Sylgard-lined 10 cm dish then fixed (4% PFA, overnight at 4°C). Tissues were washed (3 × 1 h with PBS) and stored in PBS with 0.1% sodium azide at 4°C. Longitudinal muscle-myenteric wholemounts were prepared by separating the external muscle layer from the submucosa. Circular muscle strips were then removed to expose the underlying myenteric plexus. Myenteric wholemounts were blocked and permeabilized using blocking buffer (5% normal horse serum, 0.5% Triton X-100 in PBS with 0.1% sodium azide) overnight at 4°C. Tissues were incubated for one week at 4°C with primary antibodies against PGP9.5 (rabbit, 1:2000, RRID: AB_10891773; Abcam) and HuC/D (mouse, 1:500, RRID: AB_221448; clone 16A11, Molecular Probes) diluted in blocking buffer. Tissues were then washed with PBS (3 × 30 min), then incubated with secondary antibodies conjugated to Alexa Fluor 488, 568, 594, or 647 (1:500 dilution in PBS, overnight, 4°C; ThermoFisher). Tissues were stained with DAPI (1:1000, 2 h at RT; Sigma Aldrich), washed (3 × 30 min with PBS), then mounted onto slides using buffered glycerol. Coverslips were sealed with clear nail varnish. Images of myenteric ganglia were acquired using a Leica TCS-SP8 laser scanning confocal system (HC PL APO CS2 20x/0.75 NA immersion objective) and Leica Application Suite X software (v3.5.5.19976, Leica Microsystems). Images capturing 3–6 different ganglia or regions were acquired per wholemount preparation for each patient. Images were excluded for analysis if ganglia appeared damaged due to dissection. As in mice, individual ganglia containing neuronal soma were manually traced using Fiji open-source software (ImageJ v1.54f^43^) and Feret’s diameters for each ganglia were calculated using the in-built measurements function in Fiji.

#### Myenteric ganglia enrichment and isolation

Mice were euthanized and the intestines collected as above. The muscularis propria containing the myenteric plexus was removed by mechanical dissection from colons on silicone-lined petri dishes, or the small bowel.^15^ Tissues were subsequently minced and digested in collagenase type XI (1 mg mL^−1^; Sigma Aldrich, St. Louis, Missouri, CAT# C7657) and dispase (0.4U mL^−1^; STEMCELL Technologies, Vancouver, Canada) solution at 37°C for 1 h using a VWR Mini Incubated Shaker (Avantor CAT# 76407–108). Samples were triturated until all the sample could pass through a 1000 mL pipette tip. The digested suspension was then serially passed through a set of sterile stackable cell strainers (pluriStrainer, pluriSelect, El Cajon, CA, USA) with a 20 μm pore size strainer on the bottom and 100 μm pore size strainer placed on top. The contents passing through both the 100 μm and 20 μm cell strainers were considered a single cell suspension (<20 μm), while the contents in the mid-section of the filters (20–100 μm) was collected to obtain tissue fragments enriched for MyGa. All contents >100 μm were discarded. Successful enrichment for MyGa was confirmed by transgenic tdTomato fluorescence in BAF53b-Cre neuronal reporter mice.

For human studies specimens were stored overnight at 4°C. Under a stereoscopic microscope, the muscularis propria was removed from the mucosa, submucosal, and serosal layers by mechanical dissection with fine forceps and blunt dissection with microdissection scissors. Prewarmed enzymatic solution of collagenase type XI (1 mg mL^−1^) and dispase (0.6U mL^−1^) in DMEM/F12 was added to tissues at 1 mL of enzymatic solution added per 200mg of tissue. Tissues were then minced into approximately 5mm pieces with sterile microdissection scissors and incubated in a VWR Mini Incubated Shaker at 37°C for 4 h. Samples were triturated using glass serological pipettes with progressively smaller bore diameters (3 mm, 2 mm, and 1 mm) until the sample became liquefied. Large undigested fragments of tissue were filtered out of the solution using a 1000 μm cell strainer (pluriStrainer, pluriSelect, El Cajon). Samples were then passed through a 100 μm cell strainer to collect the single cell suspension (<100 μm), and the remaining contents that failed to pass through (100–1000 μm) were collected to isolate the human MyGa enriched fraction. In some experiments MyGa were manually picked from this fraction under a stereoscopic microscope using fine forceps. Red blood cell removal was performed by incubating samples in ACK Lysing Buffer (Gibco, ThermoFisher Scientific) for 7 min. To validate MyGa enrichment, samples were collected in RLT buffer for gene expression analysis by PCR or 4% PFA for immunohistochemistry with anti-Tubulin β3 (TUBB3, 1:200, TUJ1 clone conjugated to Alexa Fluor 488).

#### Neurosphere generation and cell culture

Culture of neurospheres from mouse intestinal tissues was conducted in defined enteric neurosphere culture media.^44^ Mice were euthanized and the small intestine was processed as described above in *quantification of ganglia sizes in mouse samples*. Single cell suspensions (<20 μm) and enriched MyGa fragments (20–100 μm) were cultured in enteric neurosphere media for 10 days in free-floating conditions (Corning Costar 24-well Clear Flat Bottom Ultra-Low Attachment Multiple Well Plates Cat# 3473) to generate neurospheres utilized for spheroid quantification, gene expression analysis, and cell transplantation experiments. For monolayer cultures, neurospheres generated after 14 days in free-floating conditions were transferred to tissue-culture treated plates (CytoOne 24 well Plate, TC Treated) coated with fibronectin (1:100 for 2h at 37°C, Millipore Sigma Fibronectin Bovine Plasma Cat# F1141–5MG) in the same media for an additional 14 days to facilitate cell migration. Monolayer cultures were utilized for immunohistochemistry experiments to visualize and characterize EGC and enteric neuronal subpopulations and to promote the trypsinization of cultures into single cell suspensions for fluorescence-activated cell sorting (FACS) experiments.

For the culture of human cells, single cell suspensions (<100 μm), enriched MyGa fragments (100–1000 μm) and hand-picked MyGa were cultured in free-floating conditions (Corning Costar 6-well Clear Flat Bottom Ultra-Low Attachment Multiple Well Plates, Cat# 3471) in neurosphere generation media^45^ with the addition of Antibiotic-Antimycotic (Gibco, ThermoFisher Scientific) in a standard cell culture incubator (37°, 5% CO2, and atmospheric O2). Plating density was determined according to the initial tissue weight with samples plated in 1 mL of media per 250 mg of initial tissue weight. Samples were cultured for 10–14 days with the media volume doubled midway through culture to generate neurospheres (passage 0). Neurospheres were then transferred to tissue-culture treated plates (CytoOne 6 well Plate, TC Treated Cat# CC7682–7506) coated with fibronectin (1:100 for 2h at 37°C) for the formation of monolayers over 10–14 days of culture with the media completely replaced midway. This monolayer culture step facilitated the removal of non-living debris that failed to adhere to the fibronectin-coated wells and allowed samples to be trypsinized to single cell suspensions (end of passage 0). Cells were counted and reseeded at a density of 5000 cells/cm^2^ in free-floating conditions with the cell culture steps repeated as above for the first and second passages. Neurospheres were collected in RLT buffer and frozen for gene expression studies across passage 0 to 3.

#### Quantitative PCR

Quantitative PCR was performed to assess the gene expression of neural stem cell, glial and neuronal markers.^46^ Briefly, samples were transferred to a microcentrifuge tube and centrifuged at 500*g* for 5 min to remove the supernatant. Samples were resuspended in RLT buffer (Qiagen) and stored at −80°C until the time of RNA extraction using an RNeasy Mini kit (Qiagen). The concentration of extracted RNA was quantified using the Qubit RNA HS Assay Kit (Invitrogen, Thermo Fisher Scientific) on a Qubit 4 Fluorometer (Invitrogen, Thermo Fisher Scientific). Total RNA was reverse transcribed and amplified via RT-qPCR with the iTaq Universal SYBR Green One-Step Kit (Bio-Rad) using a Bio-Rad CFX96 real-time thermal cycler with the reaction setup performed as per the manufacturer’s instructions. Primer sequences for gene amplification in mouse samples are provided in Table S2.

The thermal cycling protocol was per the kit manufacturer’s instructions. Results were processed with the Bio-Rad CFX Manager software (version 3.1), with a standard threshold to quantity cross point (Ct) values. Ct values were normalized to *Gapdh* expression in each sample as an internal control and the Log2 of the fold change (FC) was calculated between corresponding sample conditions from the same mouse or subject. Reactions were performed in duplicate.

#### Immunohistochemistry *in vitro*

For mouse studies, cells in monolayer culture were fixed for 30 min with 4% PFA (Electron Microscopy Science, #15710), permeabilized with 0.1% Triton in PBS for 20 min and blocked in solution containing 10% donkey serum (Sigma, #D9663) for 1 h at room temperature. Primary antibodies were incubated in PBS containing 10% donkey serum at 4°C overnight. Cells were incubated with secondary antibodies below for 1 h in the same blocking solution at room temperature and stained with DAPI (Invitrogen, #D1306) for 10 min. Primary antibodies utilized in this study included: rabbit anti-Calretinin (1:200, Invitrogen, Thermo Fisher Scientific, 18–0211), rabbit nNOS (1:400, Invitrogen, Thermo Fisher Scientific, 61–7000), goat anti-GFAP (1:500, abcam, ab53554), rabbit anti-P75 (1:500, Millipore Sigma, AB1554) and human anti-Hu (Anna-1) positive serum (1:16,000, gifted from Dr. Vanda Lennon; Mayo Clinic). Secondary antibodies were utilized at 1:200 for donkey anti-rabbit 647, anti-goat 647 and donkey anti-human Alexa Fluor 488 (Invitrogen). The samples were washed between each step in PBS for 3 × 5min. For human studies, samples (passage 1) were cultured on fibronectin-coated tissue-culture plates (CytoOne 24 well Plate, TC Treated) for 10–14 days and were processed as above. Primary antibodies included: rat anti-CD49f (1:100, APC conjugated, BioLegend, Cat# 313616), mouse anti-CD271/NGFR (1:100, FITC conjugated, BioLegend, Cat# 345104) and mouse anti-Tubulin β 3 (1:300, Alexa Fluor 647 conjugated, BioLegend, Cat# 801210).

#### Cell population image analysis

To visualize and quantify EGC and enteric neuronal populations Plp1-EGFP; Baf53btdT dual = reporter mice were used. The immunohistochemically labeled cells and transgenic fluorescence were imaged on a Keyence BZX-700 All-In-One Microscopy system (Keyence America Itasca). Images were analyzed using Fiji open-source software (ImageJ v1.54f^43^) using a custom Fiji macro to quantify the number nuclei within the area occupied by transgenic and immunohistochemical fluorescence within a 10 mm^2^ field of view per sample.

Briefly, images of Baf53btdT and Plp1-EGFP fluorescence were binarized using the ‘Intermodes’ and ‘Max Entropy’ methods, respectively. Binary images were despeckled (removal of black dots < 2μm^2^) and the region occupied by Baf53btdT and Plp1-EGFP expressing cells was added to the region of interest manager using the ‘create selection’ function. Channels containing DAPI were then analyzed to count the number of nuclei within the Baf53btdT and Plp1-EGFP occupied region of interest to quantify the number of cells. This was automated by generating images in the DAPI channel containing only the regions of interest of Baf53btdT or Plp1-EGFP and binarizing images using the ‘Yen’ method. Images were further processed with the ‘Fill Holes’ and ‘Watershed’ functions, and the number of nuclei were quantified using the ‘Analyze Particles’ (>4μm^2^) function. Proportions of Baf53btdT or Plp1-EGFP expressing cells were compared to the number of nuclei in the whole field of the images as a reference to determine cell proportions.

Subpopulations of enteric neurons (Baf53btdT) and EGC/progenitors (Plp1-EGFP) were determined as similar to described above. Channels of immunohistochemical staining were processed to include only the Baf53btdT or Plp1-EGFP defined region of interest and were then binarizing using the ‘Max Entropy’ method. The subsequent region of interest of overlapping expression was used to define the area for nuclei quantification yielding the number of double-positive cells. This analysis was conducted in a total 10mm^2^ area.

Semi-automated quantification of antibody immunoreactivity in human cells was performed by generating regions of interest in the DAPI channel using the ‘Analyze Particles’ workflow as described above. The mean fluorescence intensity (mean gray values) for immunofluorescence for each antibody labeling was recorded for each cell and corrected for background fluorescence levels. Thresholds for positivity by corrected mean fluorescence intensity were manually determined by training on subsets of positive cells for each image with a minimum of 1400 cells included in the final analysis per sample (average: 8137 ± 2104 cells per sample).

#### Flow cytometry analysis and neurogenesis assay

Flow cytometric analysis was conducted using data generated on a BD FACSAria cell sorter (BD Biosciences, Franklin Lakes, New Jersey) using FlowJo software (FlowJo, LLC, OR). Single cell suspensions were produced by the trypsinization of monolayer cultures as described in *Neurosphere generation and cell culture.* BAF53btdT; Plp1-EGFP dual reporter mice were utilized to quantify the proportions of enteric neurons, EGC/progenitors, and double-negative cells with DAPI serving as a dead cell marker. Samples were acquired on an Aurora Cytek Spectral analyzer and data analyzed using FlowJo Treestar software.

To determine the ability of EGCs/progenitors to differentiate into enteric neurons BAF53btdT; Plp1-EGFP dual reporter mice were used. Monolayer cultures were produced as above and FACS was performed to purify the Plp1-EGFP expressing population. Plp1-EGFP cells were cultured in free-floating conditions to form neurospheres in defined enteric neurosphere culture media^44^ for 10 days before being seeded on fibronectin-coated plates and cultured for a further 4 days in the same media to facilitate cell migration and the visualization of individual cells. Cultures were imaged on a Keyence BZX-700 All-In-One Microscopy system and the number of Plp1-EGFP expressing cells and the emerging population of BAF53btdT expressing cells were manually quantified using the cell counter plugin of Fiji within a 10 mm^2^ field of view per sample.

#### Calcium imaging

For calcium imaging studies, ENSCs were generated from Baf53b-Cre mice crossed with GCaMP5-tdT mice (Baf53b-GCaMP5) mice with constitutive expression of the tdTomato reporter and the genetically encoded calcium indicator, GCaMP5, expressed by enteric neurons. ENSCs were cultured on fibronectin-coated culture plates for 10 days. Media was replaced with BrainPhys media (STEMCELL Technologies) and intracellular calcium ([Ca^2+^]_i_) transients in enteric neurons cells were recorded using the Keyence BZX-700 All-In-One Microscopy system. Analysis of intracellular calcium level was performed using ImageJ. Briefly, regions of interest (ROIs) were produced using images of tdTomato fluorescence and the mean fluorescence intensity (mean gray value) of GCaMP5 fluorescence, representative of intracellular calcium [Ca^2+^]_i_, was measured in each frame using ImageJ by calculating the change of fluorescence intensity expressed as the relative fluorescence (Δ*F*/*F*_0_).^47^ Acetylcholine was spritzed at a final concentration of 100μM, and electric field stimulation (EFS) was delivered by two parallel silver/platinum electrodes using a CS4 constant voltage stimulator with MyoPulse software (Danish Myo Technology) at a single pulse of 40–80 V and 0.2 ms pulse duration.

Calcium imaging in human cell monolayer cultures (passage 2) was performed using the Fluo-4 Calcium Imaging Kit (Invitrogen, Thermo Fisher Scientific, MA, #F10489) according to the manufacturers protocol using a BZX-700 All-In-One Microscope. Acetylcholine was spritzed at a final concentration of 100μM with or without a 10 min preincubation with the voltage-gated sodium channel blocker tetrodotoxin (TTX, 1μM). Recordings were processed in ImageJ and R studio. First, regions of interest were set for each cell in the field of view by creating maximum intensity projections from all video frames. These images containing elevated fluorescence in all cells at baseline or after stimulation were binarized, had the watershed function applied, and a particle analysis was performed using *Fiji*. These regions of interest were used to measure the mean fluorescence intensity of Fluo-4 across the video recordings for each individual cell. Data were normalized to Δ*F*/*F*_0_ as described in mice and matrices of Δ*F*/*F*_0_ values for cells over time were processed using the *ComplexHeatmap* package^48^ in R studio with modifications by the *circlize* and *Pals* packages.

#### Cell transplantation

To examine the engraftment, spread, and differentiation of mouse MyGa-derived neurospheres, neurospheres were generated from BAF53btdT; Plp1-EGFP dual reporter mice and transplanted to 3 month old colorless wildtype mice. Recipient mice were anesthetized by isoflurane inhalation with a midline abdominal incision made to expose the mid-colon. Microinjections of neurosphere suspensions in sterile PBS were administered in a volume of 4 μL to the muscularis propria of the colon with 100 neurospheres delivered to each recipient. Optogenetics studies were performed to assess neuronal function from transplanted MyGa-derived neurospheres by delivering cells isolated from ChAT-Cre or Baf53b-Cre mice crossed with ROSA26-tdTomato-channelrhodopsin-2 (R26-tdT-ChR2) mice. In these experiments neurospheres were produced and delivered in the same manner as above to Plp1-EGFP recipients to confirm integration of transplants with the recipient ENS.

For human ENSC transplantation studies, cells cultured in monolayer during the first passage were transduced with an Adeno-Associated Virus Type 6 carrying channelrhodopsin-2 (ChR2) and a GFP reporter driven off a CAG promoter (AAV6-CAG-ChR2-GFP; provided by Dr. Edward S Boyden, Massachusetts Institute of Technology, and produced by the UNC Vector Core, University of North Carolina).^49^ Cells were transduced at a multiplicity of infection (MOI) of 10,000 for 72h before the media was replaced and fluorescence of the GFP reporter was confirmed after one week. Monolayers were then trypsinized and taken to the second passage in free-floating conditions as described in *Neurosphere generation and cell culture*. After 10 days, human-derived neurospheres were administered to the midcolon of immunodeficient NOD-*scid* IL2Rgamma^null^ (NSG) mice as described above. For immunohistochemical and functional optogenetic studies mice were sacrificed 3 weeks after transplantation.

#### Immunohistochemistry of transplanted cells *in vivo*

Tissues were blocked and permeabilized in solution containing 10% donkey serum, 10% BSA and 1% Triton in PBS for 1 h at room temperature on a rocking platform. Primary antibodies anti-Tubulin β3 (TUBB3, 1:200, TUJ1 clone conjugated to Alexa Fluor 647) or rabbit anti-P75 (1:500, Millipore Sigma AB1554) were diluted in the same buffer and incubated overnight at 4°C before washing tissues in PBS. For P75 labeling, the secondary antibody donkey anti-rabbit Alexa Fluor 647 (1:500, Thermo Fisher Scientific, MA) was applied for 3 h in blocking buffer (PBS with 10% donkey serum and 10% BSA) before nuclei staining with DAPI, washing in PBS, and mounting onto glass slides with AquaPolymount (Polysciences). Imaging was conducted on a Keyence BZX-700 All-In-One Microscopy system (Keyence America Itasca) and a Nikon AXR confocal microscope (Nikon Cambridge, MA).

#### Smooth muscle activity recordings

To assess colonic smooth muscle activity in mice *in vivo,* electromyography (EMG) and intraluminal pressure were assessed simultaneously.^41^ Briefly, mice were subjected to anesthesia by isoflurane with a midline incision made to access the colon for ligation and cannulation with a pressure transducer (CWE Inc., Ardmore, PA). The colon was then filled with physiological saline to 10–15 mmHg in a closed system with increases in luminal pressure signifying colonic contractions. For EMG recordings, custom-made three-lead needle electrodes (Motion Lab Systems, Inc. LA, CA) were positioned in the muscularis propria of the colon and connected to a four channel Bio-amplifier (CWE Inc., Ardmore, PA) through an ISO-Z Isolated Head Stage amplifier (CWE Inc., Ardmore, PA). Digitized recordings were made with the Power Lab 16/35 data acquisition system (ADInstruments, NSW, Australia) and data were analyzed with Lab Chart Pro Software v8.1.16 (ADInstruments). Blue light stimulation of transplanted ChR2 expressing neurons was applied at 25mW, 10 ms pulse width, 10 Hz, with a 20 s train duration using a 200 μm diameter optic fiber and a diode-pumped solid-state laser system (470 nm, 200 mW, Model number: MDL-III-470; OptoEngine, LLC, Midvale, UT). For organ bath experiments, the contractile force of the colonic smooth muscle was recorded in response to blue light stimulation.^41,42^ Colons were excised and cut into 5mm rings under a fluorescent stereoscopic microscope with the presence of transplanted cells verified by tdT fluorescence for mouse-derived neurospheres, or GFP fluorescence for human-derived neurospheres. Colonic rings were mounted to force displacement transducers in a muscle strip myograph bath (Model 820 MS; Danish Myo Technology, Aarhus, Denmark) containing Krebbs solution (oxygenated with 95% O2 and 5% CO2) and were maintained at 37°C. Contractile responses were recorded with Lab Chart Pro Software and blue light stimulation was performed as above at 25mW, 10 ms pulse width, 10 Hz, with a 15 s train duration. To confirm blue light stimulation evoked contractions were neurally-mediated, tissue preparations were incubated with 1μM tetrodotoxin (TTX) for 5 min to block voltage-gated sodium channels.

#### Single cell RSNA-seq

Human cells in monolayer culture at the end of the second passage were trypsinized and cell counts performed. cDNA libraries were prepared using the Chromium Single Cell 3ʹ Reagent Kit v3.1 (10X Genomics, CA) using the standard 10X Genomics workflow according to manufacturer’s instructions and sequenced on an Illumina NextSeq platform (Illumina, Inc., CA) at the MGH NextGen Sequencing Core Facility. Output data were processed using the 10X genomics Cell Ranger Count v7.1.0 pipeline using the human reference genome (GRCh38) to generate barcode and feature count matrices for downstream analysis.^50^ Data were analyzed using the *Seurat* package in the RStudio integrated development environment.^51^ Data were preprocessed by corrections for ambient RNA using *soupX*,^52^ removing ribosomal, heat shock protein, immediate-early, and sex specific genes. High quality cells were included with between 600 and 5000 unique features, greater than 1000 UMI counts, and less than 15% of mitochondrial RNA. Doublet detection was performed using the R package *DoubletFinder*.^53^ Data was processed using the standard Seurat v5 workflow with the functions SCTransform(), FindVariableFeatures() and RunPCA(). Cell cycles were evaluated in each cell using the CellCycleScoring function of Seurat and were treated as variables to regress. RPCAIntegration was performed across samples using the IntegrateLayers() and JoinLayers() functions. Nearest neighbors were calculated and UMAPs were generated by the FindNeighbors() and RunUMAP() commands with reduction method set to “RPCA” with 1:30 dimensions. Clusters were determined using FindClusters() and the resolution was optimized for each *in silico* experiment based on the maximum number of unique clusters with supporting literature, bona fide markers, and further experimental validation. Visualization of cell annotations or gene expression in UMAP space and Dotplots were performed using Seurat with modifications by the packages ggplot2, viridis, and pals. Differentially expressed genes (DEGs) between clusters or treatments was performed using the FindAllMarkers function with a minimum log2FC value of 0.5 and adjusted *p* value <0.05. Mouse enteric neurosphere data was obtained from Guyer et al. (GSE184981)^27^ and processed using Seurat as above. Human EGC data was obtained from the Broad Institute Single Cell Portal (study number SCP1038) and processed as above using original authors’ annotations for non-subject specific glial populations.^34^ Human neurosphere cell population markers were assessed in data from mouse neurospheres^27^ and human EGCs^34^ using the *AddModuleScore* function to identify corresponding cell populations.

### QUANTIFICATION AND STATISTICAL ANALYSIS

#### Statistical analysis

All details of statistical analysis can be found in the figure legends and Data S1. Data analysis was performed using GraphPad Prism v7 (GraphPad Software Inc., San Diego, USA). For all analyses *p* ≤ 0.05 was considered significant. All data were presented as mean ± standard error of the mean (SEM), unless otherwise stated.

## Supplementary Material

1

2

3

4

5

6

7

## Figures and Tables

**Table:** KEY RESOURCES TABLE

REAGENT or RESOURCE	SOURCE	IDENTIFIER
Antibodies		
Anti-Calretinin	Thermo Fisher Scientific	18–0211; RRID:AB_2925239
Anti-nNOS	Thermo Fisher Scientific	61–7000; RRID: AB_2313734
Anti-GFAP	Abcam	Ab53554; RRID:AB_880202
Anti-Nerve Growth Factor Receptor, p75	Millipore Sigma	Ab1554; RRID:AB_11211656
Anti-Hu (ANNA-1)	gifted from Dr. Vanda Lennon; Mayo Clinic	RRID:AB_2314657
Anti-human/mouse CD49f conjugated to APC	Biolegend	313616; RRID: AB_1575047
Anti-human CD271 (NGFR) conjugated to FITC	Biolegend	345104; RRID:AB_2282828
Anti-TUBB3 conjugated to Alexa Fluor 647	Biolegend	801210; RRID: AB_2686930
Anti-TUBB3 conjugated to Alexa Fluor 488	Biolegend	801203; RRID:AB_2564757
Anti-PGP9.5	Abcam	Ab108986; RRID:AB_10891773
Anti-Human HuC/HuD	Molecular Probes	A-21271; RRID:AB_221448
Donkey anti-rabbit Alexa Fluor 647	Thermo Fisher Scientific	A-31573; RRID:AB_2536183
Donkey anti-goat Alexa Fluor 647	Thermo Fisher Scientific	21447; RRID:AB_2535864
Donkey anti-human DyLight^™^ 488	Thermo Fisher Scientific	SA5–10126; RRID:AB_2556706
Bacterial and virus strains		
AAV6-CAG-ChR2-GFP	provided by Dr. Edward S Boyden, Massachusetts Institute of Technology, and produced by the UNC Vector Core, University of North Carolina	Addgene Plasmid #26929
Biological samples		
Human intestinal specimens	Massachusetts General Hospital, Boston, USA	Table S1
Specimens of ganglionated colon taken from patients with HSCR (4–21 months of age) or ARM (9–20 months of age)	Royal Children’s Hospital, Melbourne, Australia	N/A
Chemicals, peptides, and recombinant proteins		
Diphtheria toxin	Sigma Aldrich	DO564
DAPI (4ʹ,6-Diamidino-2-Phenylindole, Dihydrochloride)	Invitrogen	D1306
Critical commercial assays		
Chromium Single Cell 3ʹ Reagent Kit v3.1	10X Genomics	PN-1000128
Deposited data		
Single-cell RNA sequencing data (Neurospheres)	This paper	Dataverse https://doi.org/10.7910/DVN/SWKB0S
Experimental models: organisms/strains		
Mouse: BAF53b-Cre	JAX Stock #027826	Mature post-mitotic neuron specific Cre^31,40^
Mouse: Plp1-EGFP	Kindly gifted by Wendy Macklin PhD, U Colorado	GFP fluorescent reporter for *Plp1* (glia)^29^
Mouse: ROSA26-tdTomato (R26-tdT)	JAX Stock #007914	Cre dependent tdT fluorescent reporter^15^
Mouse: GCaMP5g-tdTomato (GCaMP5-tdT)	JAX Stock #024477	Genetically encoded calcium indicator and tdT fluorescent reporter for Cre^15^
Mouse: ROSA26-tdTomato-channelrhodopsin-2 (R26-tdT-ChR2)	JAX Stock #012567	Cre-dependent expression of channelrhodopsin^32,40,41^
Mouse: ChAT-Cre	JAX Stock #031661	Cholinergic neuron specific Cre^41^
Mouse: NOD-*scid* IL2Rgamma^null^ (NSG)	JAX Stock #005557	Immunodeficient for humanization and xenotransplantation
Oligonucleotides		
Primer sequences for PCR	This study	Table S2
Software and algorithms		
Seurat	Satija Lab, New York Genome Center, USA	https://satijalab.org/seurat/
ImageJ	National Institutes of Health, USA	https://imagej.nih.gov/ij/
GraphPad Prism v7	GraphPad Software, San Diego, USA	https://www.graphpad.com/scientific-software/prism/
MyoPulse software	Danish Myo Technology, Aarhus, Denmark	https://www.dmt.dk/myoview.html
Lab Chart Pro Software v8.1.16	ADInstruments NSW, Australia	https://www.adinstruments.com/support/software
FlowJo software	FlowJo, LLC, OR, USA	https://www.flowjo.com/solutions/flowjo
Fiji macro for neuronal and glial subpopulation counting *in vitro*	This study	https://doi.org/10.5281/zenodo.13844556

## Highlights

- Glia in the MyGa have higher neurogenic potential than intramuscular glia do
- MyGa can be isolated by their enzymatic resistance and counter filtration
- Enteric neural stem cells (ENSCs) cultured from MyGa improve neuronal differentiation
- Human MyGa-derived cells engraft and exert neuromuscular connectivity in mice
