# Supplementary material for: Highly neurogenic glia from human and mouse myenteric ganglia generate functional neurons following culture and transplantation into the gut
Source: Cell Rep. Author manuscript; Available in PMC 2025 Jan 3. (PMC11697211; doi:10.1016/j.celrep.2024.114919)
Supplement: 1 [file NIHMS2038907-supplement-1.pdf]

**Cell Reports, Volume *43***

## Supplemental information

# Highly neurogenic glia from human and mouse myenteric ganglia generate functional neurons following culture and transplantation into the gut

**Jessica L. Mueller, Abigail R. Leavitt, Ahmed A. Rahman, Christopher Y. Han, Leah C. Ott, Narges S. Mahdavian, Simona E. Carbone, Sebastian K. King, Alan J. Burns, Daniel P. Poole, Ryo Hotta, Allan M. Goldstein, and Rhian Stavely**

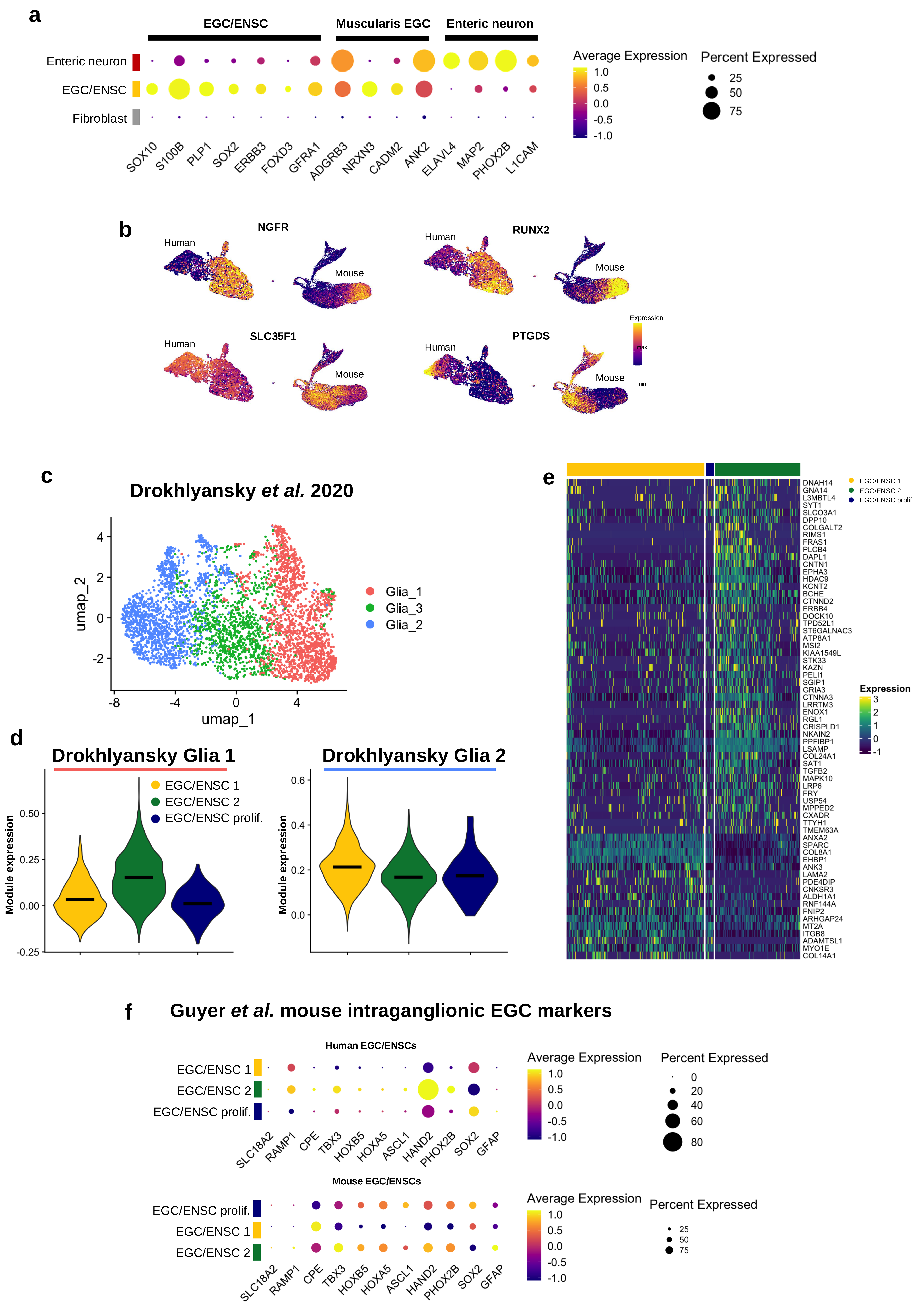
a
EGC/ENSC
Muscularis EGC
Enteric neuron
Enteric neuron
EGC/ENSC
Fibroblast
SOX10
S100B
PLP1
SOX2
ERBB3
FOXD3
GFRA1
ADGRB3
NRXN3
CADM2
ANK2
ELAVL4
MAP2
PHOX2B
L1CAM
Average Expression
1.0
0.5
0.0
-0.5
-1.0
Percent Expressed
25
50
75
b
NGFR
Human
Mouse
RUNX2
Human
Mouse
SLC35F1
Human
Mouse
PTGDS
Human
Mouse
Expression
max
min
c
Drokhlyansky et al. 2020
umap_2
umap_1
4
2
0
-2
-8
-4
0
4
Glia_1
Glia_3
Glia_2
d
Drokhlyansky Glia 1
EGC/ENSC 1
EGC/ENSC 2
EGC/ENSC prolif.
Module expression
0.50
0.25
0.00
-0.25
Drokhlyansky Glia 2
Module expression
0.6
0.4
0.2
0.0
e
EGC/ENSC 1
EGC/ENSC 2
EGC/ENSC prolif.
DNAH14
GNA14
L3MBTL4
SYT1
SLCO3A1
DPP10
COLGALT2
RIMS1
FRAS1
PLCB4
DAPL1
CNTN1
EPHA3
HDAC9
KCNT2
BCHE
CTNND2
ERBB4
DOCK10
TPD52L1
ST6GALNAC3
ATP8A1
MSI2
KIAA1549L
STK33
KAZN
PELI1
SGIP1
GRIA3
CTNNA3
LRRTM3
ENOX1
RGL1
CRISPLD1
NKAIN2
PPFIBP1
LSAMP
COL24A1
SAT1
TGFB2
MAPK10
LRP6
FRY
USP54
MPPED2
CXADR
TTYH1
TMEM63A
ANXA2
SPARC
COL8A1
EHBP1
ANK3
LAMA2
PDE4DIP
CNKSR3
ALDH1A1
RNF144A
FNIP2
ARHGAP24
MT2A
ITGB8
ADAMTSL1
MYO1E
COL14A1
Expression
3
2
1
0
-1
f
Guyer et al. mouse intraganglionic EGC markers
Human EGC/ENSCs
EGC/ENSC 1
EGC/ENSC 2
EGC/ENSC prolif.
SLC18A2
RAMP1
CPE
TBX3
HOXB5
HOXA5
ASCL1
HAND2
PHOX2B
SOX2
GFAP
Average Expression
1.0
0.5
0.0
-0.5
-1.0
Percent Expressed
0
20
40
60
80
Mouse EGC/ENSCs
EGC/ENSC prolif.
EGC/ENSC 1
EGC/ENSC 2
SLC18A2
RAMP1
CPE
TBX3
HOXB5
HOXA5
ASCL1
HAND2
PHOX2B
SOX2
GFAP
Average Expression
1.0
0.5
0.0
-0.5
-1.0
Percent Expressed
25
50
75

**Figure S1. Additional scRNA-seq analysis. Related to Figure 6.**

**a)** Dotplot visualization of markers for EGC/ENSCs, muscularis EGCs and enteric neurons in human cultures. **b)** UMAP visualization of gene expression markers for human (left) and mouse (right) EGC/ENSC 1 and 2 subpopulations. **c)** UMAP visualization of glia clusters from human intestine defined by Drokhlyansky et al. 2020. **d-e)** Module scoring of human EGC/ENSC 1 and 2 markers in human glia populations. **f)** Expression of intraganglionic EGC markers defined by Guyer et al. in human and mouse EGC/ENSC cultures.

**Table S1.** Details of subjects utilized for cell isolation. Related to Figure 6.

| Study ID | Sample | Age (years) | Sex | Type of Surgery | Diagnosis |
|---|---|---|---|---|---|
| **1** | Colon | 17 | Female | Colectomy | Familial adenomatous polyposis |
| **2** | Colon | 61 | Female | Ileocolectomy | Crohn's disease with complication, unspecified gastrointestinal tract location |
| **3** | Cecum and Ileum | 22 | Male | Laparoscopic assisted ileocecal resection | Crohn’s disease stricture |
| **4** | Colon | 15 | Male | Subtotal colectomy | Chronic pseudo-obstruction |
| **5** | Colon | 15 | Male | Proctocolectomy with soave endorectal pull through and coloanal anastomosis | Chronic constipation |
| **6** | Ileum | 2 mos | Male | Exploratory laparotomy, bowel resection | Stricture secondary to NEC and Meckel's diverticulum |
| **7** | Sigmoid Colon | 6.5 months | Male | Colostomy closure | Imperforate anus |
| **8** | Colon | 36 | Female | Right colectomy | Unresectable colon polyp |
| **9** | Colon | 49 | Female | Colostomy closure | Cancer |

**Table S2.** Primer sequences for PCR. Related to Figures 1,2 and 6.

| Gene | Forward sequence (5’→3’) | Reverse sequence (5’→3’) |
|---|---|---|
| **Gapdh (Ms)** | AGG TCG GTG TGA ACG GAT TTG | TGT AGA CCA TGT AGT TGA GGT CA |
| **Tubb3 (Ms)** | TAG ACC CCA GCG GCA ACT AT | GTT CCA GCT TCC AAG TCC ACC |
| **Phox2b (Ms)** | GGG CTA AGT TTC GCA AGC AG | CAG TGC TGT CGG GAT CAG TG |
| **Chat (Ms)** | GAG CGA ATC GTT GGT ATG ACA A | AGG ACG ATG CCA TCA AAA GG |
| **Nos1 (Ms)** | CTG GTG AAG GAA CGG GTC AG | CCG ATC ATT GAC GGC GAG AAT |
| **Ngfr (Ms)** | CCT GGA CAG TGT TAC GTT CTC | ACA CAG GGA GCG GAC ATA CT |
| **Pdgfra (Ms)** | ATG AGA GTG AGA TCG AAG GCA | CGG CAA GGT ATG ATG GCA GAG |
| **Col1a1 (Ms)** | TAA GGG TCC CCA ATG GTG AGA | GGG TCC CTC GAC TCC TAC AT |
| **GAPDH (Hu)** | GGA GCG AGA TCC CTC CAA AAT | GGC TGT TGT CAT ACT TCT CAT GG |
| **PLP1 (Hu)** | TGC TGA TGC CAG AAT GTA TGG | GCA GAT GGA CAG AAG GTT GGA |
| **NGFR (Hu)** | CCG TTG GAT TAC ACG GTC CAC | TGA AGG CTA TGT AGG CCA CAA |
| **TUBB3 (Hu)** | GGC CAA GGG TCA CTA CAC G | GCA GTC GTT TTC ACA CTC |
| **PHOX2B (Hu)** | AAC CCG ATA AGG ACC ACT TTT G | AGA GTT TGT AAG GAA CTG CGG |
